# Covalent Bonds Reinforced Strength and Modulus of Heterocyclic Aramid Fiber by Interfacial Aminated‐MXene Nanosheets

**DOI:** 10.1002/advs.75135

**Published:** 2026-04-02

**Authors:** Boyuan Chen, Hansheng Liu, Weiheng Kong, Ke Han, Jing Fang, Xingjiang Wu, Jianhong Xu, Hao Li

**Affiliations:** ^1^ National‐Local Joint Engineering Laboratory for Energy Conservation in Chemical Process Integration and Resources Utilization School of Chemical Engineering and Technology Hebei University of Technology Tianjin P. R. China; ^2^ State Key Laboratory of Chemical Engineering and Low‐Carbon Technology Department of Chemical Engineering Tsinghua University Beijing P. R. China

**Keywords:** aminated‐MXene, high strength and modulus, interfacial amido covalent bonds, microfluidic spinning, PBIA fibers

## Abstract

Architecting high‐performance heterocyclic poly(p‐phenylene benzimidazole‐benzamide) (PBIA) fiber presents challenges, including low orientation, crystallinity and interchain interaction, which reduces mechanical property. Herein, for the first time, we develop covalent bonds strategy to reinforce the mechanical property of PBIA fiber by using interfacial aminated‐MXene (MXene‐NH_2_) nanosheets via in situ polymerization and microfluidic spinning. The MXene‐NH_2_ with reactive amino sites can serve as nucleating agents and chain anchors to enhance orientation, crystallinity and interchain interaction via strong interfacial amido covalent bonds. Consequently, the representative PBIA/Ti_3_C_2_T_x_‐NH_2_ fiber exhibits the increase of 34% in tensile strength and 18% in Young's modulus, with an ultrahigh tensile strength of 6.95 ± 0.16 GPa and Young's modulus of 273.6 ± 5.1 GPa. This covalent bonds reinforced mechanism can be further extended to other MXene‐NH_2_ nanosheets, such as V_2_CT_x_‐NH_2_, Nb_2_CT_x_‐NH_2_ and Mo_2_TiC_2_T_x_‐NH_2_, indicating the universality. Benefiting from ultrahigh strength and modulus, the PBIA/Ti_3_C_2_T_x_‐NH_2_ fiber can maintain 61% initial strength after 0.8 J transverse impact, and the CNTs@PBIA/Ti_3_C_2_T_x_‐NH_2_ fiber supercapacitor presents durable capacitance under stretching state, indicating the promising paradigm for high‐performance fiber as impact‐protection and wearable supercapacitor.

## Graphical abstract

We demonstrate the covalent bonds strategy to enhance the mechanical property of PBIA fiber by using interfacial MXene‐NH_2_ nanosheets. The MXene‐NH_2_ nanosheets with reactive amino sites that can serve as nucleating agents and chain anchors to enhance orientation, crystallinity and interchain interaction of PBIA fiber via strong interfacial amido covalent bonds. Therefore, the PBIA/MXene‐NH_2_ fiber exhibits ultrahigh strength and modulus.

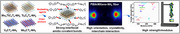

## 1 Introduction

Synthetic high‐performance fiber featuring high mechanical property and good environmental stability, together with highlighting superiorities of lightweight and facile integrating/weaving has become promising candidates in aerospace, special protection, wearable electronics and low‐altitude economy [1, 2, 3, 4, 5, 6, 7, 8]. Among them, poly(p‐phenylene benzimidazole‐benzamide) (PBIA) fiber, as a kind of heterocyclic aramid fibers, inherits flexible heterocyclic units of 2‐(4‐aminophenyl)‐5‐aminobenzimidazole (PABZ) that can regulate the polymer chains symmetry, which usually exhibits superior mechanical property and manufacturability than that of traditional high‐performance fibers [9, 10, 11, 12, 13]. However, the low crystallinity, orientation and interchain interaction will inevitably generate structural defects and rebarbative stress concentration points, and therefore produce the regret of huge gap between practical and theoretical mechanical property of PBIA fiber [14, 15, 16, 17, 18].

To date, spinning regulation and polymer chain design have been developed to enhance mechanical properties of PBIA fiber [19, 20, 21, 22, 23, 24]. In this respect, the spinning regulation primarily involves the optimization of the draw ratio. Specifically, the appropriate draw ratio during the spinning process can effectively enhances the orientation of molecular chains along the fiber axis. However, excessive stretching will reduce the toughness of the fiber, increasing the fracture risk during spinning process, and therefore suffers from insufficient improvement in mechanical properties [22, 25, 26, 27, 28]. As for polymer chain design, the molar ratio optimization of heterocyclic monomer can enhance dispersibility and processability of PBIA, facilitating the orientation improvement of fiber. However, this approach will inevitably destroy the polymer chain integrity and reduce the crystallinity, which also exhibits insufficient improvement in mechanical properties [20, 29, 30, 31].

Viewing from the unique interfacial compatibility and flexible engineering, the interfacial chemistry affords fascinating advantages to reinforce the crystallinity, orientation and interchain interaction of PBIA fiber [23, 32, 33, 34]. To this end, the interfacial hydrogen bonds have been proposed to improve mechanical properties of PBIA fiber, such as 0D SiO_2_/PBIA fiber (13% increase in tensile strength) [23], 2D RGO/PBIA fiber (tensile strength of 5.81 GPa) [14]. Nevertheless, the interfacial hydrogen bonds possess low bond energy and usually generate weak interchain interaction between PBIA chains, and therefore result in unsatisfactory reinforcement for PBIA fiber (tensile strength<5.81 GPa, Young's modulus<150 GPa) [22, 35]. Compared to weak interfacial hydrogen bonds, the interfacial covalent bonds featuring high bond energy can endow strong interchain interaction between PBIA chains, and therefore produce high reinforcement for PBIA fiber [32]. For example, the short aminated single‐walled carbon nanotube (sa‐SWNTs) can form strong interfacial covalent bonds between PBIA chains, which delivers high crystallinity and orientation degree. As a result, the sa‐SWNTs/PBIA fiber presents large tensile strength of 6.44 GPa and Young's modulus of 141.7 GPa [32]. However, the easy tangle and difficult dispersity of carbon nanotubes remain a huge challenge, and will affect the structural integrity of PBIA fiber [35, 36, 37]. Different from sa‐SWNTs, the MXene with rich surface chemistry (oxygen‐containing and fluorine‐containing groups as active sites), superior mechanical property and excellent dispersibility has been widely employed to enhance high‐performance polymers [38, 39, 40]. For example, the MXene with excellent dispersibility can enhance the tensile strength of ultrahigh molecular weight polyethylene (UHMWPE) because of abundant interchain interaction between MXene nanosheets and UHMWPE molecular chains (increased by 9%) [40]. These distinctive physicochemical and mechanical properties endow that the MXene nanosheets can serve as new‐generation interfacial enhancement for high‐performance aramid fibers. However, to date, the MXene nanosheets has never been reported to reinforce mechanical property of aramid fiber, let alone PBIA fiber. Therefore, developing MXene nanosheets to enhance the mechanical properties of PBIA fiber via strong interfacial covalent bonds is highly desired.

In this work, for the first time, the new‐type MXene‐NH_2_ nanosheets with reactive amino sites and superior dispersity are demonstrated to reinforce the mechanical property of PBIA fiber via strong interfacial amido covalent bonds. Specifically, the MXene‐NH_2_ nanosheets are in situ polymerized with PBIA monomers to form PBIA/MXene‐NH_2_ spinning dope, which is further processed into PBIA/MXene‐NH_2_ fiber via microfluidic spinning. As a typical representative, the PBIA/Ti_3_C_2_T_x_‐NH_2_ fiber delivers ultrahigh tensile strength of 6.95 ± 0.16 GPa with increase of 34% and large Young's modulus of 273.6 ± 5.1 GPa with increase of 18%. The outstanding mechanical properties are mainly attributed to that the MXene‐NH_2_ nanosheets with reactive amino sites can serve as nucleating agents and chain anchors to enhance orientation, crystallinity and interchain interaction of PBIA fiber via strong interfacial amido covalent bonds between MXene‐NH_2_ nanosheets and PBIA polymer chain. The molecular dynamics (MD) simulation, wide‐angle (WAXS), small‐angle (SAXS) x‐ray and spherical‐aberration corrected TEM (AC‐TEM) are conducted to further demonstrate the covalent bonds reinforced mechanism of PBIA/MXene‐NH_2_ fiber. Remarkably, this covalent bonds reinforced mechanism can be further extended to other MXene‐NH_2_ nanosheets, such as V_2_CT_x_‐NH_2_, Nb_2_CT_x_‐NH_2_ and Mo_2_TiC_2_T_x_‐NH_2_, indicating the universality. Based on remarkable strength and modulus, the PBIA/Ti_3_C_2_T_x_‐NH_2_ fiber maintains 61% initial strength after 0.8 J transverse impact, exhibiting the promising application in impact‐protection. Moreover, the supercapacitor assembled by CNTs@PBIA/Ti_3_C_2_T_x_‐NH_2_ fiber presents durable capacitance under stretching state. This covalent bonds reinforced strategy provides significant guidance for architecting ultrastrong aramid fiber and highlights the development of next‐generation synthetic high‐performance fiber.

## 2 Results and Discussion

### 2.1 Preparation of PBIA/MXene‐NH_2_ Fiber

Generally, the strong interfacial amido covalent bonds is greatly significant to reinforce the mechanical property of PBIA fiber [41]. The traditional interfacial enhancement methods in PBIA fibers have been widely investigated, such as 0D SiO_2_, 1D CNTs and 2D RGO. However, the 0D SiO_2_ usually suffers from less active site, and therefore provides weak interfacial hydrogen bonds between PBIA and SiO_2_ nanoparticles [23]. For 1D CNTs, the easy tangle and poor dispersity will simultaneously introduce structural defects inside PBIA fibers [32]. Although 2D RGO possesses excellent dispersibility, the onefold oxygen‐containing group as active sites usually affords weak interfacial hydrogen bonds for PBIA polymer chain [14]. As a result, the traditional interfacial enhancement methods still suffer from unsatisfactory reinforcement for PBIA fiber (modulus: <200 GPa). Notably, the 2D MXene‐NH_2_ nanosheets with reactive amino sites can serve as nucleating agents and chain anchors to enhance orientation, crystallinity and interchain interaction of PBIA fiber via strong interfacial amido covalent bonds between MXene‐NH_2_ nanosheets and PBIA polymer chain, and therefore provides high reinforcement for PBIA fiber (modulus: 273.6 GPa).

To achieve strong interfacial amido covalent bonds, the MXene‐NH_2_ nanosheets with reactive amino sites that can react with PBIA monomers are synthesized by amination reaction with ammonia water. As a typical representative, the Ti_3_C_2_T_x_ MXene nanosheets are utilized as an example to conduct the structural and chemical characterization of MXene‐NH_2_ nanosheets [42]. As shown in Figures S1 and S2, the scanning electron microscopy (SEM), transmission electron microscope (TEM), atomic force microscope (AFM), Fourier‐transform infrared (FTIR) spectroscopy, Raman spectra, x‐ray diffraction (XRD), x‐ray photoelectron spectroscopy (XPS) all demonstrate successful synthesis of MXene‐NH_2_ nanosheets. The dispersity of the Ti_3_C_2_T_x_‐NH_2_ nanosheets is investigated, as shown in Figure S1e. Notably, the prepared Ti_3_C_2_T_x_‐NH_2_ nanosheets can be stably dispersed in DMAc solvent for over seven days without any sedimentation under static conditions. Moreover, the UV spectrum analysis reveals that the Ti_3_C_2_T_x_ and Ti_3_C_2_T_x_‐NH_2_ nanosheets exhibit similar absorption curves, indicating their excellent dispersibility (Figure S1f). Furthermore, the SEM‐EDS analysis confirms that the amino groups are uniformly grafted onto the surface of MXene (Figure S3), which is crucial for the formation of interfacial covalent bonds.

**FIGURE 1:**
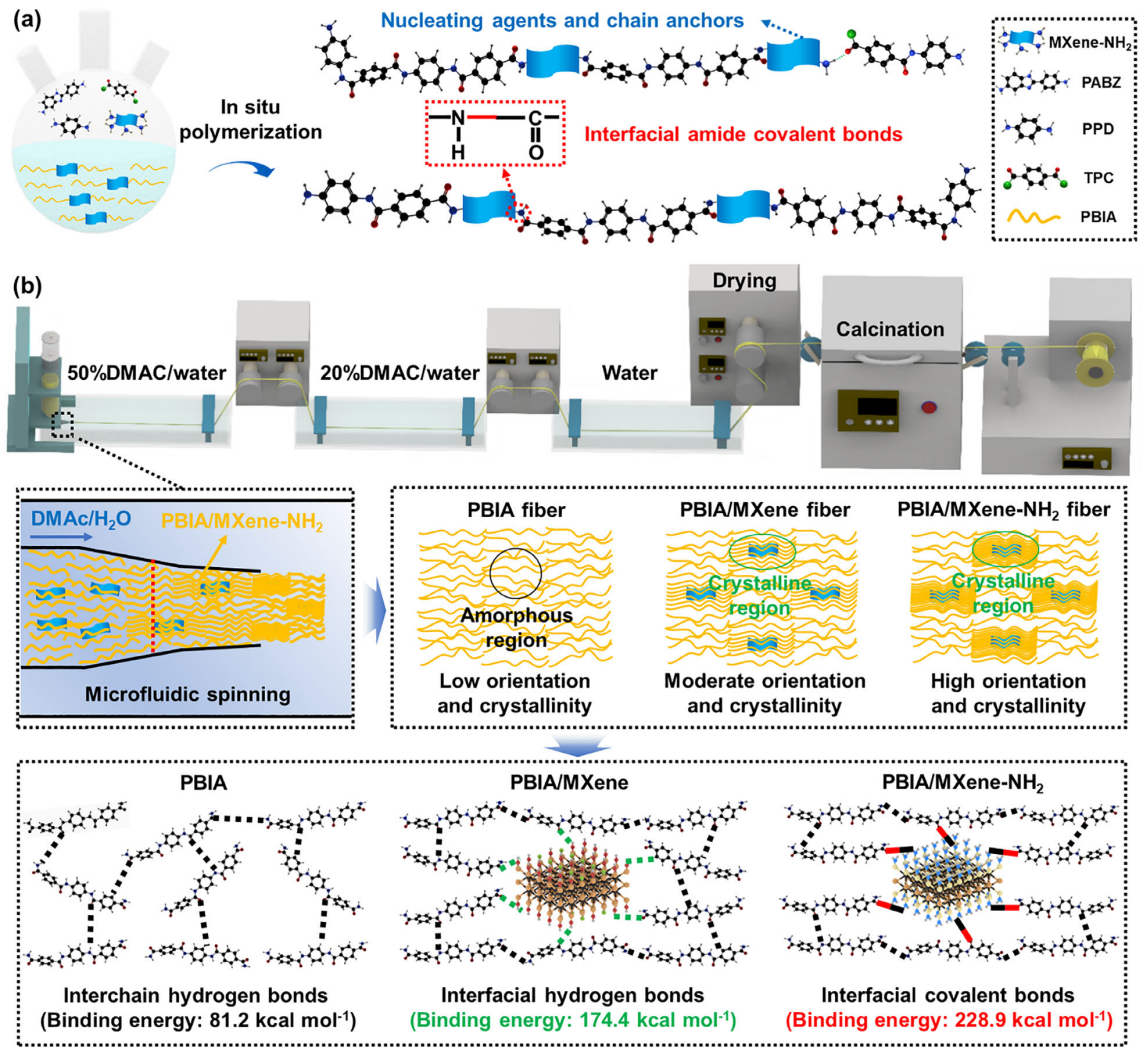
(a) Schematic in situ polymerization of PBIA/MXene‐NH_2_ spinning dope. (b) Schematic preparation of PBIA/MXene‐NH_2_ fiber via microfluidic spinning and the corresponding reinforcement mechanism.

As depicted in Figure 1a, the MXene‐NH_2_ nanosheets with active amino groups can serve as nucleating agents and chain anchors to react with terephthaloyl chloride (TPC) via amidation reaction during in situ polymerization, and therefore produce strong interfacial amido covalent bonds between MXene‐NH_2_ nanosheets and PBIA polymer chain. Therefore, the specific effects of MXene and MXene‐NH_2_ nanosheets on molecular weights of PBIA are also explored by molecular weight measurements via gel permeation chromatography (GPC). As measured in Figure S4, the PBIA/Ti_3_C_2_T_x_‐NH_2_ spinning dope presents slightly higher molecular weight (Mw = 73404 g mol^−1^) and lower polymer dispersity index (PDI = 1.9) than that of PBIA spinning dope (Mw = 70311 g mol^−1^, PDI = 2.02) and PBIA/Ti_3_C_2_T_x_ spinning dope (Mw = 72305 g mol^−1^, PDI = 2.0), affirming that Ti_3_C_2_T_x_‐NH_2_ nanosheets with reactive amino sites can facilitate the in situ polymerization of TPC, p‐phenylenediamine (PPD) and PABZ. The amidation reaction between Ti_3_C_2_T_x_‐NH_2_ nanosheets and TPC is further confirmed by FTIR spectra. As shown in Figure S5a, after amidation reaction, the TPC/Ti_3_C_2_T_x_‐NH_2_ nanosheets exhibit higher C‐N characteristic peak than that of Ti_3_C_2_T_x_‐NH_2_ nanosheets, attributing to the formation of strong interfacial amido covalent bonds from amidation reaction between TPC and Ti_3_C_2_T_x_‐NH_2_ nanosheets [43]. More notably, the C─Cl bond characteristic peak of TPC is disappeared, indicating that TPC is reacted by Ti_3_C_2_T_x_‐NH_2_ nanosheets. Furthermore, we analyzed the high‐resolution N1s XPS spectrum of the TPC/Ti_3_C_2_T_x_‐NH_2_ nanosheets. As observed in Figure S5b, compared to Ti_3_C_2_T_x_‐NH_2_ nanosheets (Figure S2e)_,_ a new O═C─NH characteristic peak of TPC/Ti_3_C_2_T_x_‐NH_2_ nanosheets is observed at 400.5 eV, attributing to the formation of strong interfacial amido covalent bonds by amidation reaction between TPC and Ti_3_C_2_T_x_‐NH_2_ nanosheets.

Subsequently, the PBIA/MXene‐NH_2_ spinning dope is processed into PBIA/MXene‐NH_2_ fiber via microfluidic spinning. As illustrated in Figure 1b, the coaxial microfluidic chip with gradually contracted microchannel can produce elongational flow with strong shearing stress along the flow direction of PBIA/MXene‐NH_2_ spinning dope, which induces the MXene‐NH_2_ nanosheets and PBIA polymer chain to form ordered arrangements from disordered arrangements, and further enhance orientation. Generally, the PBIA fiber with interchain hydrogen bonds (low binding energy: 81.2 kcal mol^−1^) usually suffers from low crystallinity and orientation degrees and interchain interaction, which produces low mechanical property. Notably, the MXene nanosheets inhering abundant oxygen‐containing/fluorine‐containing groups can serve as orientation seeds to introduce interfacial hydrogen bonds (moderate binding energy: 174.4 kcal mol^−1^) into PBIA network, and therefore improve crystallinity and orientation degrees and interchain interaction of PBIA fiber, which presents improved strength and modulus. However, both interchain hydrogen bonds and interfacial hydrogen bonds only afford weak interchain interaction for PBIA network, and therefore produce limited enhancement in mechanical property of PBIA fiber. Compared with MXene nanosheets, the MXene‐NH_2_ nanosheets with reactive amino sites can serve as nucleating agents and chain anchors to introduce interfacial amido covalent bonds (high binding energy: 228.9 kcal mol^−1^) into PBIA network. As a result, the MXene‐NH_2_ nanosheets can greatly improve crystallinity and orientation degrees and interchain interaction of PBIA fiber, which presents high strength and modulus. As a result, the MXene‐NH_2_ nanosheets can greatly improve crystallinity, orientation degrees and interchain interaction of PBIA fiber, which presents high strength and modulus (Figure S6).

### 2.2 Structural Characterization of PBIA/Ti_3_C_2_T_x_‐NH_2_ Fiber

**FIGURE 2:**
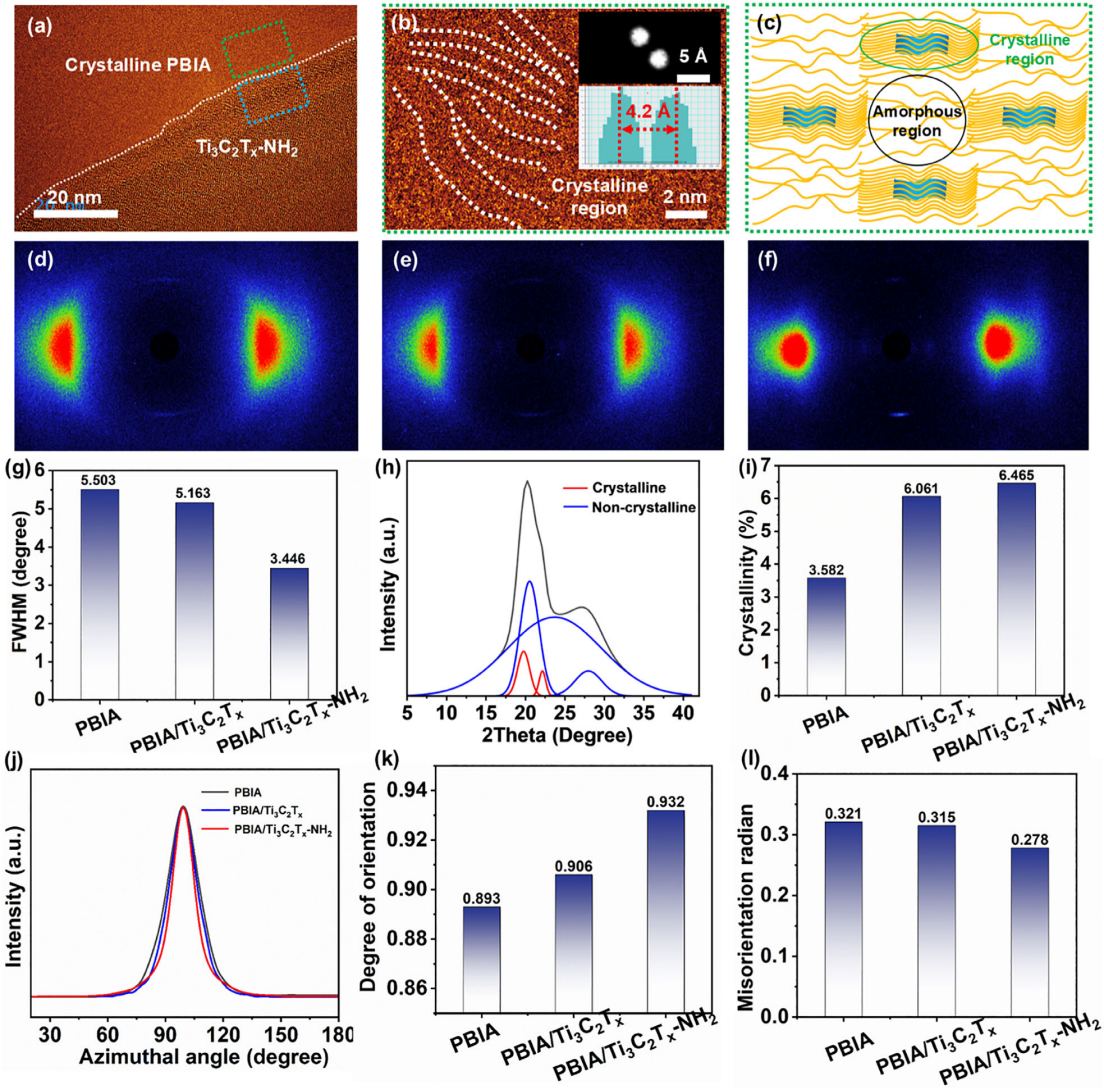
(a,b) The AC‐TEM images of PBIA/Ti_3_C_2_T_x_‐NH_2_ fiber. (c) The schematic illustration of crystalline region and amorphous region in PBIA/Ti_3_C_2_T_x_‐NH_2_ fiber. (d–f) The 2D‐WAXS of PBIA, PBIA/Ti_3_C_2_T_x_ and PBIA/Ti_3_C_2_T_x_‐NH_2_ fibers. (g) The FWHM of PBIA, PBIA/Ti_3_C_2_T_x_ and PBIA/Ti_3_C_2_T_x_‐NH_2_ fibers. (h) The 1D‐WAXS and corresponding peak fitting curves of PBIA/Ti_3_C_2_T_x_‐NH_2_ fiber. (i) The crystallinity degree of PBIA, PBIA/Ti_3_C_2_T_x_ and PBIA/Ti_3_C_2_T_x_‐NH_2_ fibers. (j) The Azimuthal intensity curves of PBIA, PBIA/Ti_3_C_2_T_x_ and PBIA/Ti_3_C_2_T_x_‐NH_2_ fibers. (k) The orientation degree of PBIA, PBIA/Ti_3_C_2_T_x_ and PBIA/Ti_3_C_2_T_x_‐NH_2_ fibers. (l) The misorientation radian of PBIA, PBIA/Ti_3_C_2_T_x_ and PBIA/Ti_3_C_2_T_x_‐NH_2_ fibers.

As typical representative, the as‐prepared PBIA, PBIA/Ti_3_C_2_T_x_ and PBIA/Ti_3_C_2_T_x_‐NH_2_ spinning dopes are continuously processed into PBIA, PBIA/Ti_3_C_2_T_x_, and PBIA/Ti_3_C_2_T_x_‐NH_2_ fibers via microfluidic spinning. The chemical structures of PBIA, PBIA/Ti_3_C_2_T_x_ and PBIA/Ti_3_C_2_T_x_‐NH_2_ fibers are characterized by FTIR spectra (Figure S7). Compared with PBIA and PBIA/Ti_3_C_2_T_x_ fibers (1652.94 cm^−1^), the C═O bond characteristic peak of PBIA/Ti_3_C_2_T_x_‐NH_2_ fiber shifts to 1654.86 cm^−1^, due to the formation of interfacial amido covalent bonds from amidation reaction between TPC and Ti_3_C_2_T_x_‐NH_2_ nanosheets. Figure S8 presents the scanning electron microscopy (SEM) images of PBIA, PBIA/Ti_3_C_2_T_x_ and PBIA/Ti_3_C_2_T_x_‐NH_2_ fibers. Obviously, the PBIA, PBIA/Ti_3_C_2_T_x_ and PBIA/Ti_3_C_2_T_x_‐NH_2_ fibers show almost same surface morphologies, suggesting that the addition of Ti_3_C_2_T_x_ and Ti_3_C_2_T_x_‐NH_2_ nanosheets not influence the fiber preparation. To reveal the crystallinity and interchain interaction of PBIA/Ti_3_C_2_T_x_‐NH_2_ fiber, the ultrathin sections prepared by focused ion beam (FIB) of PBIA/Ti_3_C_2_T_x_‐NH_2_ fiber is studied by spherical‐aberration corrected TEM (AC‐TEM) (Figure S9). As illustrated in Figure 2a, the crystalline PBIA is tightly concatenated into Ti_3_C_2_T_x_‐NH_2_ nanosheets interface, indicating the strong interfacial interaction between Ti_3_C_2_T_x_‐NH_2_ nanosheets and PBIA polymer chain. Figure 2b,c and Figure S10 deliver the specific lattice natures of PBIA and Ti_3_C_2_T_x_‐NH_2_ nanosheets. Remarkably, the PBIA exhibits abundant crystalline regions and distinct lattice fringe with a lattice spacing of 0.42 nm around the Ti_3_C_2_T_x_‐NH_2_ nanosheets, demonstrating that Ti_3_C_2_T_x_‐NH_2_ nanosheets with reactive amino sites can serve as nucleating agents to enhance crystallinity degree of PBIA fiber [32].

Moreover, the crystallinity and orientation degrees of PBIA, PBIA/Ti_3_C_2_T_x_ and PBIA/Ti_3_C_2_T_x_‐NH_2_ fibers are evaluated by wide‐angle x‐ray diffraction (WAXS). As shown in Figure 2d,e,f, the PBIA/Ti_3_C_2_T_x_‐NH_2_ fiber exhibits brighter and more focused diffraction spots near the equatorial plane than that of PBIA and PBIA/Ti_3_C_2_T_x_ fibers, implying the more ordered polymer chain arrangement in the transverse fiber direction [14]. Based on the 2D‐WAXS, the 1D‐WAXS of PBIA, PBIA/Ti_3_C_2_T_x_ and PBIA/Ti_3_C_2_T_x_‐NH_2_ fibers are obtained to analysize the full width at half maxima (FWHM) (Figure S11). As depicted in Figure 2g, the PBIA/Ti_3_C_2_T_x_‐NH_2_ fiber possesses smaller FWHM of 3.446 than that of PBIA (FWHM = 5.503) and PBIA/Ti_3_C_2_T_x_ fibers (FWHM = 5.163), manifesting the higher crystallinity degree. The specific crystallinity degree of PBIA, PBIA/Ti_3_C_2_T_x_ and PBIA/Ti_3_C_2_T_x_‐NH_2_ fibers are quantificationally calculated by 1D‐WAXS curve (Figure 2h; Figures S12 and S13). As calculated in Figure 2i, the PBIA/Ti_3_C_2_T_x_‐NH_2_ fiber yields higher crystallinity degree of 6.465 than that of PBIA (3.582) and PBIA/Ti_3_C_2_T_x_ fibers (6.061), attributing to the interfacial amido covalent bonds between Ti_3_C_2_T_x_‐NH_2_ nanosheets and PBIA polymer chain. Furthermore, the orientation degrees of PBIA, PBIA/Ti_3_C_2_T_x_ and PBIA/Ti_3_C_2_T_x_‐NH_2_ fibers are calculated by azimuthal degree of WAXS curves (Figure 2j) via following formul: $s=\frac{180-FWHM}{180}$ [22]. Notably, the PBIA/Ti_3_C_2_T_x_‐NH_2_ fiber exhibits highest orientation degree of 0.932 than that of PBIA (0.893) and PBIA/Ti_3_C_2_T_x_ fibers (0.906), validating that the orientation degree of PBIA fiber can be also enhanced by Ti_3_C_2_T_x_‐NH_2_ nanosheets (Figure 2k). In addition, the microfibrillar misorientation degree is further evaluated by using small‐angle x‐ray scattering (SAXS) and corresponding 2D‐SAXS patterns (Figures S14–S16). Thereinto, the arc between the microfibril and fiber axis is defined as the misorientation arc (𝐵𝜑), which is calculated by Ruland streak method via equatorial streak characteristics of SAXS (Figures S17–S19) [20]. Notably, the PBIA/Ti_3_C_2_T_x_‐NH_2_ fiber displays smallest arc of 0.278 than that of PBIA (0.321) and PBIA/Ti_3_C_2_T_x_ fibers (0.315), signifying that the arrangement of PBIA/Ti_3_C_2_T_x_‐NH_2_ fiber is more ordered (Figure 2l; Figure S20). Undoubtedly, the Ti_3_C_2_T_x_‐NH_2_ nanosheets with reactive amino sites can serve as nucleating agents to enhance crystallinity and orientation degrees of PBIA fiber via interfacial amido covalent bonds. Figure S21 presents the water contact angles of PBIA, PBIA/Ti_3_C_2_T_x_ and PBIA/Ti_3_C_2_T_x_‐NH_2_ fibers. Astonishingly, the PBIA/Ti_3_C_2_T_x_‐NH_2_ fiber has lower water contact angle of 75.5° and higher surface energy of 38.3 mN m^−1^ than that of PBIA (contact angle of 95.7°, surface energy of 25.7 mN m^−1^) and PBIA/Ti_3_C_2_T_x_ fibers (contact angle of 82.0°, surface energy of 34.2 mN m^−1^). This result may be mainly ascribed to the introduction of Ti_3_C_2_T_x_ and Ti_3_C_2_T_x_‐NH_2_ improved the polarity of fiber surface [43].

### 2.3 Mechanical Property of PBIA/Ti_3_C_2_T_x_‐NH_2_ Fiber

**FIGURE 3:**
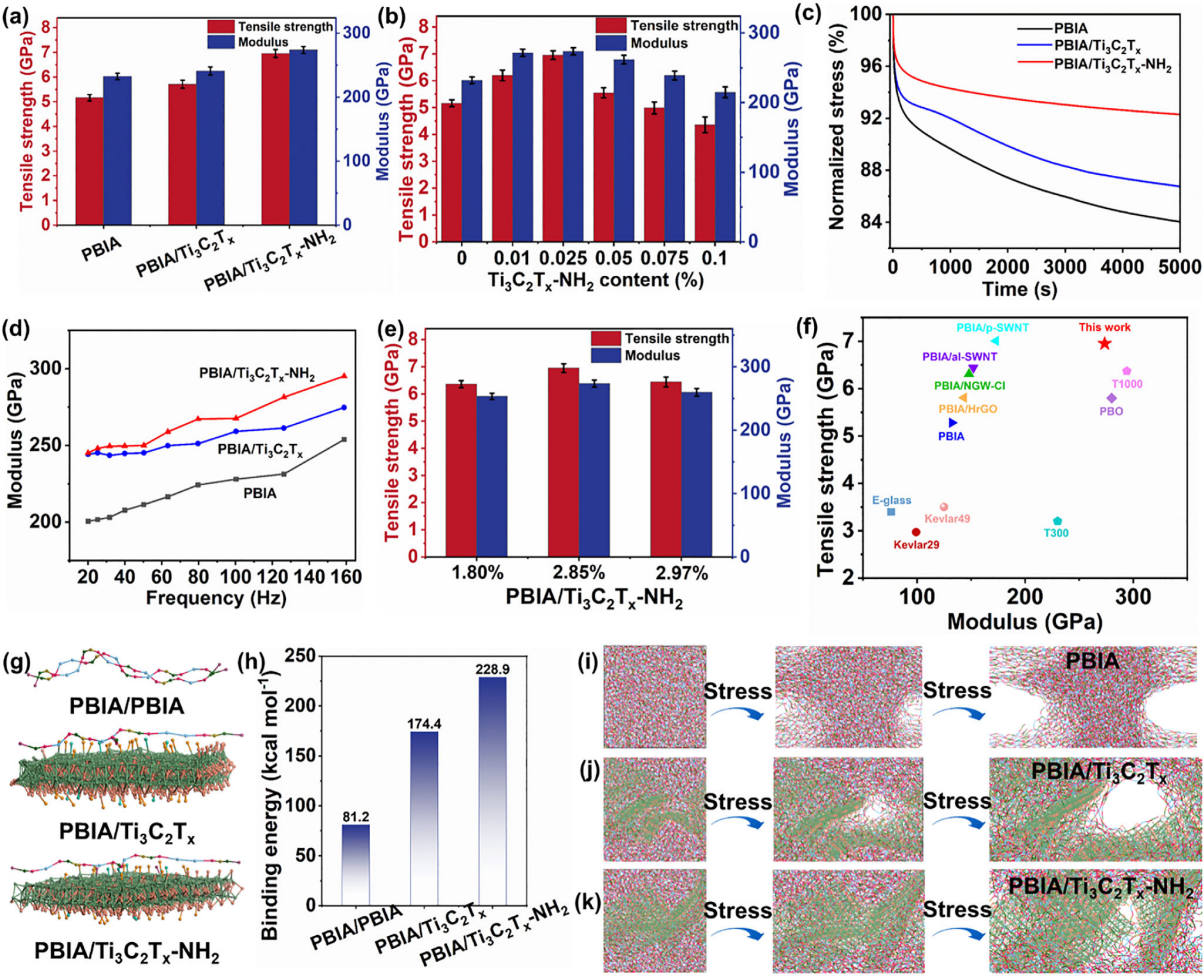
(a) The tensile strength and Young's modulus of PBIA, PBIA/Ti_3_C_2_T_x_ and PBIA/Ti_3_C_2_T_x_‐NH_2_ fibers. (b) The tensile strength and Young's modulus of PBIA/Ti_3_C_2_T_x_‐NH_2_ fibers under various Ti_3_C_2_T_x_‐NH_2_ nanosheets contents. (c) The stress relaxation curves of PBIA, PBIA/Ti_3_C_2_T_x_ and PBIA/Ti_3_C_2_T_x_‐NH_2_ fibers. (d) The dynamic modulus of PBIA, PBIA/Ti_3_C_2_T_x_ and PBIA/Ti_3_C_2_T_x_‐NH_2_ fibers. (e) The tensile strength and Young's modulus of PBIA/Ti_3_C_2_T_x_‐NH_2_ fibers under various Ti_3_C_2_T_x_‐NH_2_ nanosheets with various ‐NH_2_ contents. (f) The comparation of tensile strength and Young's modulus of PBIA/Ti_3_C_2_T_x_‐NH_2_ fiber with other reported high‐performance fibers. (g) The Coarse‐grained model of PBIA/PBIA, PBIA/Ti_3_C_2_T_x_ and PBIA/Ti_3_C_2_T_x_‐NH_2._ (h) The binding energy of PBIA/PBIA, PBIA/Ti_3_C_2_T_x_ and PBIA/Ti_3_C_2_T_x_‐NH_2_. (i–k) The microstructure simulation of PBIA, PBIA/Ti_3_C_2_T_x_ and PBIA/Ti_3_C_2_T_x_‐NH_2_ fibers under stretching process.

Considering the high crystallinity and orientation degrees, the mechanical properties of PBIA, PBIA/Ti_3_C_2_T_x_ and PBIA/Ti_3_C_2_T_x_‐NH_2_ fibers are characterized to confirm that Ti_3_C_2_T_x_‐NH_2_ nanosheets can enhance the tensile strength and Young's modulus, which are crucial parameters for evaluating the high‐performance fiber. As shown in S22, the PBIA/Ti_3_C_2_T_x_ fiber exhibits higher tensile strength of 5.71 ± 0.16 GPa than that of PBIA fiber (5.16 ± 0.13 GPa), indicating that tensile strength of PBIA/Ti_3_C_2_T_x_ fiber can be enhanced by Ti_3_C_2_T_x_ nanosheets via interfacial hydrogen bonds. After amination reaction, the Ti_3_C_2_T_x_‐NH_2_ nanosheets can generate strong interfacial amido covalent bonds between Ti_3_C_2_T_x_‐NH_2_ nanosheets and PBIA polymer chain. Therefore, the PBIA/Ti_3_C_2_T_x_‐NH_2_ fiber delivers highest tensile strength of 6.95 ± 0.16 GPa with the increase of 34% and 22% in tensile strength compared to PBIA and PBIA/Ti_3_C_2_T_x_ fibers. Meanwhile, compared with PBIA fiber with golden yellow, the PBIA/Ti_3_C_2_T_x_‐NH_2_ fiber shows darker color, implying the in situ polymerization of Ti_3_C_2_T_x_‐NH_2_ nanosheets within PBIA/Ti_3_C_2_T_x_‐NH_2_ fiber. More significantly, the PBIA/Ti_3_C_2_T_x_‐NH_2_ fiber also possesses maximum Young's modulus of 273.6 ± 5.1 GPa than that of PBIA (232.4 ± 4.9 GPa) and PBIA/Ti_3_C_2_T_x_ fibers (240.9 ± 6.2 GPa) (Figure 3a, Tables S1 and S2). Figure 3b presents the tensile strength and Young's modulus of PBIA/Ti_3_C_2_T_x_‐NH_2_ fibers under various Ti_3_C_2_T_x_‐NH_2_ nanosheets contents. Notably, with the increase of Ti_3_C_2_T_x_‐NH_2_ nanosheets from 0, 0.01 to 0.025 wt.%, the tensile strength and Young's modulus increase from 5.16 ± 0.13 GPa/232.4 ± 4.9 GPa, 6.2 ± 0.2 GPa/271.5 ± 5.2 GPa to 6.95 ± 0.16 GPa/273.6 ± 5.1 GPa, respectively. However, when the Ti_3_C_2_T_x_‐NH_2_ nanosheets further increase from 0.025 to 0.05, 0.075, 0.1 wt.%, the tensile strength and Young's modulus decrease from 6.95 ± 0.16 GPa/273.6 ± 5.1 GPa to 5.55 ± 0.19 GPa/261.8 ± 6.1 GPa to 4.99 ± 0.22 GPa/238.8 ± 6.3 GPa, 4.36 ± 0.29 GPa/214.8 ± 7.8 GPa, respectively. This result suggests that small addition of Ti_3_C_2_T_x_‐NH_2_ nanosheets with good dispersion can greatly enhance the mechanical properties of PBIA fiber because of the improved crystallinity and orientation degrees. On the contrary, the excessive addition of Ti_3_C_2_T_x_‐NH_2_ nanosheets may increase the misorientation and defect sites of PBIA fiber due to the interlayer re‐stacking and slippage phenomenon [44], and therefore decrease the mechanical property. Considering the complex application conditions of high‐performance fibers, we conducted bending fatigue tests to measure the environmental stability of PBIA fiber and PBIA/Ti_3_C_2_T_x_‐NH_2_ fiber, including bending fatigue tests, knot strength measurements and humidity aging test. Notably, the PBIA/Ti_3_C_2_T_x_‐NH_2_ fiber retains 92.4% initial tensile strength after bending fatigue tests (1000 cycles), which is higher than PBIA fiber (90.3%) (Figure S23). Furthermore, after knot strength test, the PBIA/Ti_3_C_2_T_x_‐NH_2_ fiber exhibits higher strength retention (35.4% initial tensile strength) than that of PBIA fiber (30.4%) (Figure S24). Moreover, the PBIA/Ti_3_C_2_T_x_‐NH_2_ fiber also presents higher strength retention (92.9% initial tensile strength) than that of PBIA fiber (90.3%) after hydrothermal aging test (70 °C water, 20d) (Figure S25). Those outstanding results demonstrate that the PBIA/Ti_3_C_2_T_x_‐NH_2_ fiber possesses superior wearability, durability and environmental stability, due to high orientation, crystallinity and interchain interactions based on strong interfacial amido covalent bonds.

Moreover, the stress relaxation measurement of PBIA, PBIA/Ti_3_C_2_T_x_ and PBIA/Ti_3_C_2_T_x_‐NH_2_ fibers are performed to verify the interfacial interaction. As illustrated in Figure 3c, the PBIA/Ti_3_C_2_T_x_‐NH_2_ fiber still retains 92.3% initial stress after 5000 s, which is higher than that of PBIA (84%) and PBIA/Ti_3_C_2_T_x_ fiber (86.7%), confirming that Ti_3_C_2_T_x_‐NH_2_ nanosheets can reinforce the interfacial interchain interaction to prevent interchain slippage via interfacial amido covalent bonds. The dynamic modulus of PBIA, PBIA/Ti_3_C_2_T_x_ and PBIA/Ti_3_C_2_T_x_‐NH_2_ fibers are also evaluated under harmonic loads at 20–160 Hz to assess mechanical property during stretching processes. As depicted in Figure 3d, the dynamic modulus of PBIA, PBIA/Ti_3_C_2_T_x_ and PBIA/Ti_3_C_2_T_x_‐NH_2_ fibers are 253.8, 274.4, and 295.2 GPa at 160 Hz, respectively. Shatteringly, the highest dynamic modulus of PBIA/Ti_3_C_2_T_x_‐NH_2_ fiber delivers superior antiseismic performance than that of PBIA/Ti_3_C_2_T_x_ and PBIA/Ti_3_C_2_T_x_‐NH_2_ fibers, attributing to the increased loss modulus under forced vibration [14]. To further explore the effect of reactive amino sites on mechanical property of PBIA/Ti_3_C_2_T_x_‐NH_2_ fiber, the Ti_3_C_2_T_x_‐NH_2_ nanosheets with various ‐NH_2_ contents are synthesized under various ammonia water concentration to prepare the PBIA/Ti_3_C_2_T_x_‐NH_2_ fibers, such as PBIA/Ti_3_C_2_T_x_‐NH_2_‐1.80%, PBIA/Ti_3_C_2_T_x_‐NH_2_‐2.85% and PBIA/Ti_3_C_2_T_x_‐NH_2_‐2.97% (Figures S26 and S27). Interestingly, the PBIA/Ti_3_C_2_T_x_‐NH_2_‐1.80% fiber presents relatively lower mechanical property (tensile strength of 6.36 GPa and Young's modulus of 253.4 GPa) than that of PBIA/Ti_3_C_2_T_x_‐NH_2_‐2.85% fiber (tensile strength of 6.95 GPa and Young's modulus of 273.6 GPa), indicating that the low ‐NH_2_ content provides insufficient amido covalent bonds between Ti_3_C_2_T_x_‐NH_2_ nanosheets and PBIA polymer chain, and therefore produce low reinforcement efficiency. Although the higher ‐NH_2_ content can provides more amido covalent bonds between Ti_3_C_2_T_x_‐NH_2_ nanosheets and PBIA polymer chain, the PBIA/Ti_3_C_2_T_x_‐NH_2_‐2.97% fiber exhibits decreased mechanical property (tensile strength of 6.44 GPa and Young's modulus of 259.7 GPa) compared to PBIA/Ti_3_C_2_T_x_‐NH_2_‐2.85% fiber. This phenomenon mainly ascribes to the decreased dispersity and interlayer re‐stacking of Ti_3_C_2_T_x_‐NH_2_ nanosheets prepared by excess ammonia water concentration (Figure 3e; Figures S28 and S29), which may generate more defect sites within PBIA/Ti_3_C_2_T_x_‐NH_2_‐2.97% fiber. Significantly, the dazzling tensile strength (6.95 ± 0.16 GPa) and Young's modulus (273.6 ± 5.1 GPa) of PBIA/Ti_3_C_2_T_x_‐NH_2_ fiber are much higher than that of reported aramid fibers, such as Kevlar29 fiber (tensile strength of 2.97 GPa, Young's modulus of 99.1 GPa), Kevlar49 fiber (tensile strength of 3.5 GPa, Young's modulus of 125 GPa) [45], PBIA fiber (tensile strength of 5.28 GPa, Young's modulus of 132.2 GPa), PBIA/HrGO fiber (tensile strength of 5.81 GPa, Young's modulus of 143.2 GPa) [14], PBIA/NGW‐Cl fiber (tensile strength of 6.31 GPa, Young's modulus of 152 GPa) [24], and PBIA/al‐SWNT fiber (tensile strength of 6.44 GPa, Young's modulus of 141.7 GPa) (Figure 3f) [32].

The molecular dynamics (MD) simulations are performed to verify the reinforcement mechanism of Ti_3_C_2_T_x_‐NH_2_ nanosheets on PBIA/Ti_3_C_2_T_x_‐NH_2_ fiber. As illustrated in Figure 3g,h, the binding energy of PBIA/PBIA, PBIA/Ti_3_C_2_T_x_ and PBIA/Ti_3_C_2_T_x_‐NH_2_ are first calculated to confirm the interchain interaction. Notably, the PBIA/Ti_3_C_2_T_x_‐NH_2_ exhibits highest binding energy of 228.9 kcal mol^−1^ than that of PBIA/PBIA (81.2 kcal mol^−1^), PBIA/Ti_3_C_2_T_x_ (174.4 kcal mol^−1^), attributing to the interfacial amido covalent bonds between Ti_3_C_2_T_x_‐NH_2_ nanosheets and PBIA polymer chain, which can reinforce the interfacial interchain interaction to prevent interchain slippage of PBIA/Ti_3_C_2_T_x_‐NH_2_ fiber. To further validate the superior mechanical property of PBIA/Ti_3_C_2_T_x_‐NH_2_ fiber, the micristructure variation under stretching process are also calculated. As depicted in Figure 3i, the PBIA fiber suffers from severe structural defects during stretching process because of low binding energy between PBIA chains via weak interchain hydrogen bonds. Due to the enhanced binding energy between PBIA chains and Ti_3_C_2_T_x_ nanosheets via interfacial hydrogen bonds, the structural defects of PBIA/Ti_3_C_2_T_x_ fiber during stretching process are obviously relieved (Figure 3j). Compared to Ti_3_C_2_T_x_ nanosheets, the Ti_3_C_2_T_x_‐NH_2_ nanosheets with reactive amino sites can serve as nucleating agents and chain anchors, which can provide high binding energy between PBIA chains and Ti_3_C_2_T_x_‐NH_2_ nanosheets via interfacial amide covalent bonds. As a result, the PBIA/Ti_3_C_2_T_x_‐NH_2_ fiber presents negligible structural defects during stretching process (Figure 3k). The above‐mentioned results demonstrate that the strong interchain interaction via interfacial amido covalent bonds can effectively decrease structural defects and therefore enhance the mechanical property of PBIA fiber.

### 2.4 Universality of MXene‐NH_2_ Nanosheets in Reinforcement of PBIA Fiber

**FIGURE 4:**
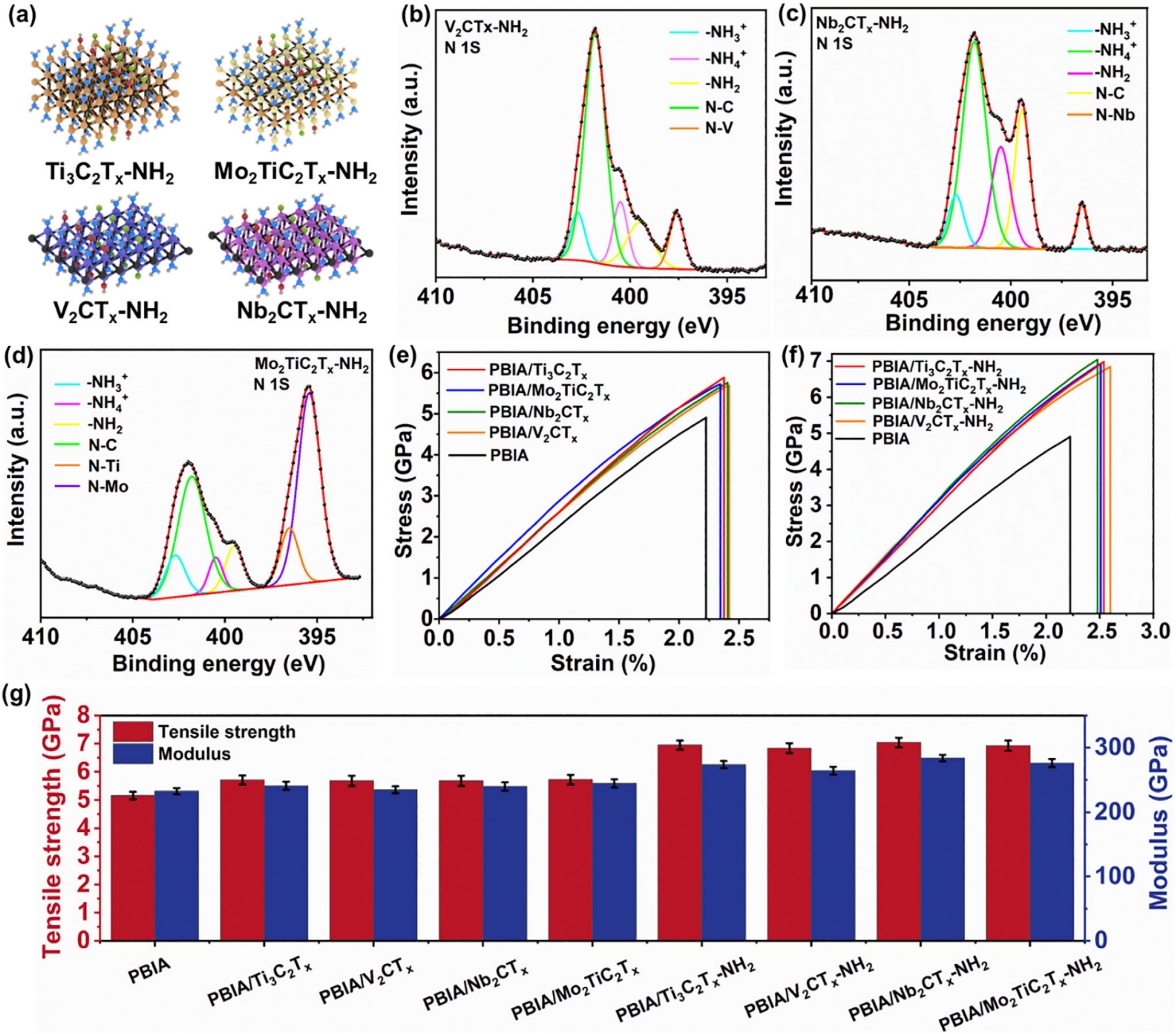
(a) The schematic illustration of Ti_3_C_2_T_x_‐NH_2_, V_2_CT_x_‐NH_2_, Nb_2_CT_x_‐NH_2_ and Mo_2_TiC_2_T_x_‐NH_2_ nanosheets. (b) The high‐resolution N 1s spectrum of V_2_CT_x_‐NH_2_ nanosheets. (c) The high‐resolution N 1s spectrum of Nb_2_CT_x_‐NH_2_ nanosheets. (d) The high‐resolution N 1s spectrum of Mo_2_TiC_2_T_x_‐NH_2_ nanosheets. (e) The stress‐strain curves of PBIA, PBIA/Ti_3_C_2_T_x_, PBIA/V_2_CT_x_, PBIA/Nb_2_CT_x_ and PBIA/Mo_2_TiC_2_T_x_ fibers. (f) The stress–strain curves of PBIA, PBIA/Ti_3_C_2_T_x_‐NH_2_, PBIA/V_2_CT_x_‐NH_2_, PBIA/Nb_2_CT_x_‐NH_2_ and PBIA/Mo_2_TiC_2_T_x_‐NH_2_ fibers. (g) The tensile strength and Young's modulus of PBIA, PBIA/Ti_3_C_2_T_x_, PBIA/V_2_CT_x_, PBIA/Nb_2_CT_x_, PBIA/Mo_2_TiC_2_T_x_, PBIA/Ti_3_C_2_T_x_‐NH_2_, PBIA/V_2_CT_x_‐NH_2_, PBIA/Nb_2_CT_x_‐NH_2_ and PBIA/Mo_2_TiC_2_T_x_‐NH_2_ fibers.

To investigate the universality of MXene‐NH_2_ nanosheets in reinforcement of PBIA fiber, a variety of MXene‐NH_2_ nanosheets are synthesized via amination reaction, such as Ti_3_C_2_T_x_‐NH_2_, V_2_CT_x_‐NH_2_, Nb_2_CT_x_‐NH_2_ and Mo_2_TiC_2_T_x_‐NH_2_ nanosheets (Figure 4a). As shown in Figure S30, compared to V_2_CT_x_, Nb_2_CT_x_ and Mo_2_TiC_2_T_x_ nanosheets, the XPS survey spectra of V_2_CT_x_‐NH_2_, Nb_2_CT_x_‐NH_2_ and Mo_2_TiC_2_T_x_‐NH_2_ nanosheets emerge new characteristic peaks of N elements, indicating the successful grafting of ‐NH_2_ groups on MXene nanosheets surface. The high‐resolution N 1s spectra of V_2_CT_x_‐NH_2_, Nb_2_CT_x_‐NH_2_ and Mo_2_TiC_2_T_x_‐NH_2_ nanosheets are also measured to confirm the presence of ‐NH_2_ groups. As illustrated in Figure 4b, the V_2_CT_x_‐NH_2_ nanosheets present characteristic peaks at 397.6, 399.2, 400.4, 401.8 and 402.8 eV, corresponding to N─V, ─NH_2_, ─NH_4_^+^, N─C, and ─NH_3_^+^, respectively. Analogously, the characteristic peaks at 396.5, 399.2, 400.4, 401.8 and 402.8 eV, belonging to N─Nb, ─NH_2_, ─NH_4_^+^, N─C, and ─NH_3_^+^ are appeared in N 1s spectra of Nb_2_CT_x_‐NH_2_ nanosheets (Figure 4c). Moreover, the N 1s spectra of Mo_2_TiC_2_T_x_‐NH_2_ nanosheets have six characteristic peaks at 395.4, 396.4, 399.2, 400.4, 401.8 and 402.8 eV, deriving from N─Mo, N─Ti, ─NH_2_, ─NH_4_^+^, N─C, and ─NH_3_^+^, respectively (Figure 4d). Those results demonstrate that the V_2_CT_x_‐NH_2_, Nb_2_CT_x_‐NH_2_ and Mo_2_TiC_2_T_x_‐NH_2_ nanosheets can be successfully synthesized via amination reaction.

Subsequently, by in situ polymerization and microfluidic spinning, the V_2_CT_x_, Nb_2_CT_x_, Mo_2_TiC_2_T_x_, V_2_CT_x_‐NH_2_, Nb_2_CT_x_‐NH_2_ and Mo_2_TiC_2_T_x_‐NH_2_ nanosheets (mass fraction of 0.025wt.%) are utilized to reinforce mechanical properties of PBIA fibers, such as PBIA/V_2_CT_x_, PBIA/Nb_2_CT_x_, PBIA/Mo_2_TiC_2_T_x_, PBIA/V_2_CT_x_‐NH_2_, PBIA/Nb_2_CT_x_‐NH_2_ and PBIA/Mo_2_TiC_2_T_x_‐NH_2_ fibers. Figure 4e shows the stress‐strain curves of PBIA, PBIA/Ti_3_C_2_T_x_, PBIA/V_2_CT_x_, PBIA/Nb_2_CT_x_ and PBIA/Mo_2_TiC_2_T_x_ fibers. Due to the interfacial hydrogen bonds between MXene nanosheets and PBIA polymer chain, the PBIA/V_2_CT_x_, PBIA/Nb_2_CT_x_ and PBIA/Mo_2_TiC_2_T_x_ fibers deliver enhanced tensile strength of 5.68, 5.75, and 5.72 GPa, respectively. Remarkably, the PBIA/V_2_CT_x_‐NH_2_, PBIA/Nb_2_CT_x_‐NH_2_ and PBIA/Mo_2_TiC_2_T_x_‐NH_2_ fibers exhibit higher tensile strength of 6.84, 7.04, and 6.93 GPa than that of PBIA/V_2_CT_x_, PBIA/Nb_2_CT_x_ and PBIA/Mo_2_TiC_2_T_x_ fibers (Figure 4f). More importantly, the PBIA/V_2_CT_x_‐NH_2_, PBIA/Nb_2_CT_x_‐NH_2_ and PBIA/Mo_2_TiC_2_T_x_‐NH_2_ fibers also present larger Young's modulus of 264.1, 283.9, and 276.1 GPa than that of PBIA (232.4 GPa), PBIA/V_2_CT_x_ (234.7 GPa), PBIA/Nb_2_CT_x_ (239.6 GPa) and PBIA/Mo_2_TiC_2_T_x_ fibers (244.4 GPa) (Figure 4g). The enhanced tensile strength and Young's modulus mainly attribute to the interfacial amido covalent bonds between MXene‐NH_2_ nanosheets and PBIA polymer chain. Based on aforesaid analyses, we can conclude that universal MXene‐NH_2_ nanosheets, such as Ti_3_C_2_T_x_‐NH_2_, V_2_CT_x_‐NH_2_, Nb_2_CT_x_‐NH_2_ and Mo_2_TiC_2_T_x_‐NH_2_ nanosheets, can serve as nucleating agents and chain anchors to enhance crystallinity and orientation degrees and interchain interaction of PBIA fiber, and therefore improve mechanical property.

### 2.5 Impact‐Protection and Wearable Fiber‐Shaped Supercapacitor Based on PBIA/Ti_3_C_2_T_x_‐NH_2_ Fiber

**FIGURE 5:**
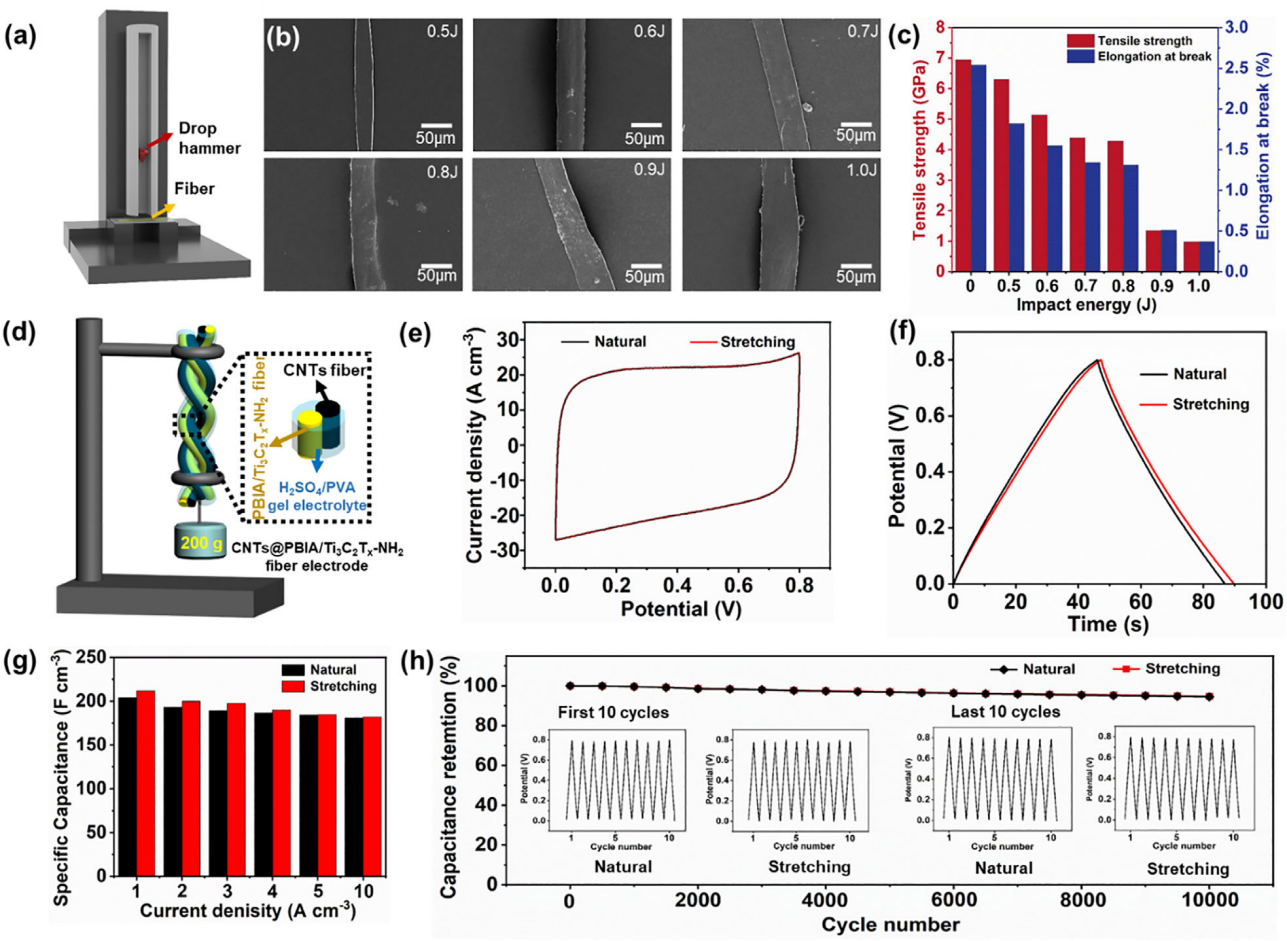
(a) The schematic diagram of the drop‐hammer test. (b) The SEM images of PBIA/Ti_3_C_2_T_x_‐NH_2_ fibers under various impact energies. (c) The tensile strength and elongation of PBIA/Ti_3_C_2_T_x_‐NH_2_ fibers under various impact energies. (d) The schematic diagram of CNTs@PBIA/Ti_3_C_2_T_x_‐NH_2_ FSC under stretching states. (e) The CV curves of CNTs@PBIA/Ti_3_C_2_T_x_‐NH_2_ FSC under natural and stretching states. (f) The GCD curves of CNTs@PBIA/Ti_3_C_2_T_x_‐NH_2_ FSC under natural and stretching states. (g) The specific capacitances of CNTs@PBIA/Ti_3_C_2_T_x_‐NH_2_ FSC under natural and stretching states. (h) The cycle stabilities of CNTs@PBIA/Ti_3_C_2_T_x_‐NH_2_ FSC under natural and stretching states.

To evaluate impact‐protection performance, the drop‐weight impact tests with graded impact energies of 0.5–1.0 J by controlling the drop hammer are conducted in Figure 5a. Notably, when the impact energy increases from 0.5 to 1 J, the PBIA/Ti_3_C_2_T_x_‐NH_2_ fibers undergo progressive flattening but have no discernible surface cracks, evidencing the high strength and modulus (Figure 5b). Figure 5c displays mechanical properties measurements of PBIA/Ti_3_C_2_T_x_‐NH_2_ fibers before and after drop‐weight impact tests. When the impact energy increases from 0.5 to 0.8 J, the tensile strength decreases from 6.95 to 4.28 GPa (61% initial strength), and the elongation decreases from 2.54% to 1.31%, respectively. Remarkably, although the impact energy further increases from 0.8 to 1 J, the PBIA/Ti_3_C_2_T_x_‐NH_2_ fiber still maintains high tensile strength (0.98 GPa) and elongation (0.37%), demonstrating the promising application in impact‐protection field.

On the basis of ultrahigh strength and modulus of PBIA/MXene‐NH_2_ fiber, the CNTs fiber is twisted with representative PBIA/Ti_3_C_2_T_x_‐NH_2_ fiber to prepare CNTs@PBIA/Ti_3_C_2_T_x_‐NH_2_ fiber, which can serve as promising electrode materials in wearable fiber‐shaped supercapacitor (FSC). As shown in Figure 5d, the FSC contains two intertwined CNTs@PBIA/Ti_3_C_2_T_x_‐NH_2_ fibers covered by H_2_SO_4_/PVA gel electrolyte, where the CNTs fiber with good electrical conductivity and abundant charge‐storage sites, and PBIA/Ti_3_C_2_T_x_‐NH_2_ fiber with ultrahigh tensile strength and Young's modulus can endow FSC with excellent energy‐storage and mechanical properties. The energy‐storage and mechanical properties of CNTs@PBIA/Ti_3_C_2_T_x_‐NH_2_ FSC are assessed by electrochemical measurements under 0.2 kg stretching. As illustrated in Figure S31, the ion dynamics characteristics of CNTs@PBIA/Ti_3_C_2_T_x_‐NH_2_ FSC under natural and stretching states are first investigated by electrochemical impedance spectroscopy (EIS) [46, 47, 48]. Interestingly, compared to natural state (C_1_/R_1_ = 0.071 mF/93.42 Ω, Zw = 80.80 Ω, τ_0_ = 14 ms), the CNTs@PBIA/Ti_3_C_2_T_x_‐NH_2_ FSC presents lower lower contact impedance (C_1_/R_1_ = 0.079 mF/84.24 Ω), diffusion impedance (Zw = 53.52 Ω) and relaxation time (τ_0_ = 9 ms) under stretching state, indicating the faster ions diffusion dynamics. The enhanced ions diffusion behavior mainly attributes to the shorter diffusion pathway because of the tighter contact between CNTs@PBIA/Ti_3_C_2_T_x_‐NH_2_ fiber and H_2_SO_4_/PVA gel electrolyte under stretching state [49]. Figure 5e presents the cyclic voltammetry (CV) curves of CNTs@PBIA/Ti_3_C_2_T_x_‐NH_2_ FSC under natural and stretching states. Noteworthily, the CV curves deliver rectangular shapes with amost the same integral areas, revealing the stable electric double‐layer capacitance (EDLC) under stretching state. Moreover, the unaltered linear relationship between scan rates and charge/discharge currents under natural and stretching states further demonstrate the excellent power and deformation‐resistance performances (Figure S32) [50]. As depicted in Figure 5f, the galvanostatic charge/discharge (GCD) curves exhibit symmetric triangular shape and amost same discharge times under natural and stretching states, further confirming the excellent ions reversibility and deformation‐resistance properties. Based on the GCD curves, the specific capacitances are calculated in Figure 5g. Obviously, the CNTs@PBIA/Ti_3_C_2_T_x_‐NH_2_ FSCs possess high specific capacitances of 204 and 212 F cm^−3^ at 1 A g^−1^ under natural and stretching states, respectively. Although current densities increase to 10 A g^−1^, the high specific capacitances of 181 and 182 F cm^−3^ under natural and stretching states are still maintained. This phenomenon manifests that the PBIA/Ti_3_C_2_T_x_‐NH_2_ fiber with high strength and modulus can effectively prevent the deformation and fracture of CNTs fiber, and therefore maintain the high specific capacitance of CNTs@PBIA/Ti_3_C_2_T_x_‐NH_2_ FSCs under stretching state. The energy density and power density are key parameters that determine the practical power‐supply application. Accordingly, we calculate the energy density and power density of CNTs@PBIA/Ti_3_C_2_T_X_‐NH_2_ fiber‐shaped supercapacitor under both natural and stretching states. Notably, the CNTs@PBIA/Ti_3_C_2_T_X_‐NH_2_ fiber‐shaped supercapacitor delivers high energy density of 4.53 mWh cm^−3^ and power density of 399.7 mW cm^−3^. This energy density value is higher than that of other wearable energy devices, such as PPy/rGO/Alg fiber (2.15 mWh cm^−3^) [51], N‐MoO_3−x_ fiber (2.29 mWh cm^−3^) [52], MXene/CNT fiber (2.55 mWh cm^−3^) [53] and MnO_2_/Ti_3_C_2_T*_x_*/RGO fiber (2.13 mWh cm^−3^) [54]. More importantly, under stretching state, the CNTs@PBIA/Ti_3_C_2_T_X_‐NH_2_ fiber‐shaped supercapacitor still delivers high energy density of 4.71 mWh cm^−3^ and power density of 397.1 mW cm^−3^, indicating the excellent mechanical properties. In addition, the cycling stabilities of CNTs@PBIA/Ti_3_C_2_T_x_‐NH_2_ FSCs are further investigated via GCD curves at 10 A g^−1^. As measured in Figure 5h, after 10 000 cycles, the CNTs@PBIA/Ti_3_C_2_T_x_‐NH_2_ FSCs can retain 94.5% and 94.7% initial capacitance under natural and stretching states, respectively, revealing the good cycling stability and mechanical property [55].

## 3 Conclusion

In summary, we develop high‐performance PBIA/Ti_3_C_2_T_x_‐NH_2_ fiber by covalent bonds strategy via in situ polymerization and microfluidic spinning, which exhibits ultrahigh tensile strength of 6.95 ± 0.16 GPa with increase of 34% and large Young's modulus of 273.6 ± 5.1 GPa with increase of 18%. The MD simulation, WAXS, SAXS and AC‐TEM reveal reinforcement mechanism that MXene‐NH_2_ nanosheets with reactive amino sites can serve as nucleating agents and chain anchors to enhance crystallinity and orientation degrees and interchain interaction of PBIA fiber via strong interfacial amido covalent bonds. Remarkably, this covalent bonds reinforced mechanism can be further extended to other MXene‐NH_2_ nanosheets, such as V_2_CT_x_‐NH_2_, Nb_2_CT_x_‐NH_2_ and Mo_2_TiC_2_T_x_‐NH_2_, indicating the universality. The superior strength and modulus can endow PBIA/Ti_3_C_2_T_x_‐NH_2_ fiber with excellent impact‐protection performance and CNTs@PBIA/Ti_3_C_2_T_x_‐NH_2_ FSCs with durable capacitance under stretching states, which exhibits promising applications in impact‐protection and wearable supercapacitor.

## 4 Experimental Methods

### 4.1 Materials

N, N‐dimethylacetamide (DMAc, 99%), p‐phenylenediamine (PPD, 99%), terephthaloyl chloride (TPC, 99%), lithium chloride (LiCl, 99%) and lithium fluoride (LiF, 99.9%) were provided by Shanghai Macklin Biochemical Co., Ltd. 2‐(4‐aminophenyl)‐5‐aminobenzimidazole (PABZ, 98%) was provided by Meryer (Shanghai) Chemical Technology Co., Ltd. Hydrochloric acid (HCl, 36%–38%) was provided by Tianjin Damao Chemical Reagent Factory. The Ti_3_AlC_2_ powder (600 mesh, 99.9%) was purchased from Jilin 11 Technology Co., Ltd. Lithium chloride was dried at 400°C for 4 h. N,N‐dimethylacetamide (DMAc) was dehydrated by using 4Å molecular sieves. Other reagents were used as received without further purification.

### 4.2 Synthesis of MXene Nanosheets

As a representative MXene, the Ti_3_C_2_T_x_ nanosheets were synthesized by acid etching method. First, 1 g LiF was dispersed in 20 mL hydrochloric acid (9 m) under magnetically stirring at 35°C for 30 min. Then, 1 g Ti_3_AlC_2_ powder was added to above solution under continuous stirring at 35°C for 48 h. After etching reaction, the mixed solution was repeatedly centrifuged and washed (deionized water) until the pH = *7*. At last, the Ti_3_C_2_T_x_ nanosheets were obtained by freeze drying treatment. The other MXene nanosheets, such as V_2_CT_x_, Nb_2_CT_x_ and Mo_2_TiC_2_T_x_ nanosheets, were directly purchased from Jilin 11 Technology Co., Ltd.

### 4.3 Synthesis of MXene‐NH_2_ Nanosheets

First, 1 g MXene nanosheets were dispersed in 50 mL deionized water. Then, the ammonia water was slowly added into MXene nanosheets dispersion under magnetically stirring until the pH = 10. Subsequently, the mixed solution was continuously stirred at 70°C for 12 h to achieve the amination reaction. After cooling to room temperature, the mixed solution was repeatedly centrifuged and washed (deionized water) until the pH = *7*. At last, the MXene‐NH_2_ nanosheets were obtained by freeze drying treatment.

### 4.4 Preparation of PBIA/MXene‐NH_2_ Spinning Dopes via in Situ Polymerization

The PBIA/MXene‐NH_2_ spinning dopes were prepared by low‐temperature polycondensation via in situ polymerization. Specifically, 1.08 g p‐phenylenediamine (PPD) and 3.36 g 2‐(4‐aminophenyl)‐5‐aminobenzimidazole (PABZ) were dispersed in 100 mL N,N‐dimethylacetamide (DMAc)/LiCl mixed solution by continuous stirring for 0.5 h. Then, a certain amount of MXene‐NH_2_ nanosheets were added into above mixed solution under continuous stirring for 10 min. Afterward, the mixed solution temperature was cooled to 0°C–5°C, and the 5.09 g terephthaloyl chloride (TPC) was slowly added into above mixed solution under continuous stirring for 2 h. After reaction, the PBIA/MXene‐NH_2_ spinning dopes with viscosity of 50 000—80 000 mPa·s were obtained.

### 4.5 Preparation of PBIA/MXene‐NH_2_ Fiber via Microfluidic Spinning

The coaxial microfluidic chip contains gradually contracted microchannel as inner phase (inner diameter of 100 µm) and cylinder microchannel as outer phase (inner diameter of 500 µm, water/DMAc mixed solution). Then, the PBIA/MXene‐NH_2_ spinning dopes was injected into inner phase by high‐pressure air pump under 0.8 MPa, and the water/DMAc coagulation solution (1:1 (v/v)) was injected into outer phase by microfluidic pump under volume flow rate of 50 mL h^−1^ to form PBIA/MXene‐NH_2_ gel fiber, which was collected in primary coagulation bath (water/DMAc (1:1 (v/v)). Subsequently, the PBIA/MXene‐NH_2_ gel fiber was drawn in a secondary coagulation bath (water/DMAc (4:1 (v/v)), and the draw ratio was 2.5. After water washing and drying treatment, the PBIA/MXene‐NH_2_ fiber was obtained and further collected via automatic winding device. The PBIA fiber and PBIA/MXene fiber were prepared by same procedures.

### 4.6 Construction of CNTs@PBIA/Ti_3_C_2_T_x_‐NH_2_ Fiber‐Shaped Supercapacitor

The CNTs@PBIA/Ti_3_C_2_T_x_‐NH_2_ fiber was prepared by twisting CNTs fiber (diameter of 60 µm, Jiangsu XFNANO Materials Tech Co., Ltd) and PBIA/MXene‐NH_2_ fiber. Then, the CNTs@PBIA/Ti_3_C_2_T_x_‐NH_2_ fiber was coated by H_2_SO_4_/PVA gel electrolyte to form electrolyte‐coated CNTs@PBIA/Ti_3_C_2_T_x_‐NH_2_ fiber. After drying at room temperature, two electrolyte‐coated CNTs@PBIA/Ti_3_C_2_T_x_‐NH_2_ fibers were twisted into CNTs@PBIA/Ti_3_C_2_T_x_‐NH_2_ fiber‐shaped supercapacitor, which was repeatedly coated by H_2_SO_4_/PVA gel electrolyte. The electrochemical measurements of CNTs@PBIA/Ti_3_C_2_T_x_‐NH_2_ fiber‐shaped supercapacitor was conducted by CHI760E electrochemical work station, such as CV, GCD and EIS curves. The specific capacitances were calculated by formula of $C\;=\frac{4\times I\times t}{\Delta V}$, where the I (A cm^−3^), 𝛥𝑡 (s) and 𝛥𝑉 (V) are current density, discharge time and operating voltage, respectively.

### 4.7 Computational Details

Coarse‐grained molecular dynamics (CGMD) simulations were conducted within the Materials Studio 2023 software utilizing the Mesocite module in conjunction with the MS Martini forcefield. An integrated approach combining Patterns, Motion Groups, and Subunits was employed for the construction of the coarse‐grained model, wherein Motion Groups was defined through the identification of specified units in the all‐atom model via the Modify section. The Bead Typing.std table was generated and validated through the Preview function, with Update performed to rectify any compositional gaps. Structural matching was further refined using Patterns encoded in Study Table documents, while the Subunits was specifically applied to polymeric systems characterized by repetitive motifs. A comprehensive set of bead types was parameterized as follows: C_6_H_4_ (76.1, 1.94, SC1), C_7_H_4_N_2_ (116, 2.31, SC5), C_8_H_4_O_2_ (132, 2.88, C1), F (19, 0.93, C1), NH_2_ (16, 1.03, Nd), NH (15, 1.01, SNda), OH (17, 0.99, P2), Ti (47.9, 0.93, C1), and TiC (59.9, 1.27, C3), with mass parameters derived from elemental summation and radius determined through atomic best‐fit spherical approximations. The simulation environment was configured through Mesostructure Template‐generated simulation boxes, subsequently populated with molecular chains and 2D architectures. Geometry Optimization was implemented via parameters including medium quality standards, Smart algorithm, 2000 maximum iterations, and isothermal conditions at 298.15 K. Subsequently, Dynamics were employed via NPT ensemble regulated by Nose thermostat and Berendsen barostat, utilizing 1 fs timestep across 200 ps to achieve convergent stability in energy, volume, and density. The equilibrated system was subjected to stress‐strain interrogation to resolve mechanical configurations under progressive deformation states.

## Funding

This work was supported by the National Natural Science Foundation of China (22478094, 22208190). Yanzhao Huangjintai Talent Plan Backbone Project (Education Platform) of Hebei Province (HJYB202507). Technology R&D Platform Special Project of Hebei Province (25361502D).

## Conflicts of Interest

None of the authors have a conflict of interest to disclose

## Supporting information




**Supporting File**: advs75135‐sup‐0001‐SuppMat.docx.

## Data Availability

The data that support the findings of this study are available from the corresponding author upon reasonable request.
