# Supplementary material for: Covalent Bonds Reinforced Strength and Modulus of Heterocyclic Aramid Fiber by Interfacial Aminated‐MXene Nanosheets
Source: Adv Sci (Weinh). 2026 Apr 2;13(36):e75135. doi: 10.1002/advs.75135 (PMC13317593; doi:10.1002/advs.75135)
Supplement: Supplementary file 1 — Supporting File: advs75135‐sup‐0001‐SuppMat.docx. [file ADVS-13-e75135-s001.docx]

Supporting Information

**Covalent Bonds Reinforced Strength and Modulus of Heterocyclic Aramid Fiber by Interfacial Aminated-MXene Nanosheets**

Boyuan Chen^[a]^, Hansheng Liu^[a]^, Weiheng Kong^[a]^, Ke Han^[a]^, Jing Fang^[a]^, Xingjiang Wu*^[a]^, Jianhong Xu^[b]^ and Hao Li*^[a]^

[a] National-Local Joint Engineering Laboratory for Energy Conservation in Chemical Process Integration and Resources Utilization, School of Chemical Engineering and Technology, Hebei University of Technology, Tianjin 300401, China.

E-mail: [wuxingjiang@hebut.edu.cn](mailto:wuxingjiang@hebut.edu.cn), [ctstlihao@hebut.edu.cn](mailto:ctstlihao@hebut.edu.cn)

[b] State Key Laboratory of Chemical Engineering and Low-Carbon Technology, Department of Chemical Engineering, Tsinghua University, Beijing 100084, P. R. China

**Characterization**

**Microscopic image characterization**

Scanning electron microscopy (SEM) images were taken with Nova Nano SEM450 field emission, the acceleration voltage is 5 KV. Transmission electron microscopy (TEM) images were recorded using a Talos F200S operating at 200 kV. Atomic Force Microscope (AFM) images were taken with Bruker Dimension icon. Axial cross-section of PBIA/Ti_3_C_2_T_x_-NH_2_ cut by focused ion beam (FIB) using Zeiss Crossbeam 550. Spherical Aberration-Corrected Transmission Electron Microscope (AC-TEM) images were recorded using Titan Themis Z.

**Multi-modal structural and compositional analysis (IR, XRD, XPS)**

Fourier Transform infrared spectroscopy (FTIR) were obtained with Vertex 80V. Raman spectra were obtained with inVia Reflex. The excitation wavelength is 532 nm, and the detector has a resolution of 576×384 pixels. X-ray diffraction (XRD) analysis was used D8 Discover. The 2θ rotation range is 5° to 50°, with a maximum power of 3 kW. X-ray photoelectron spectroscopy (XPS) test was performed using an X-ray photoelectron spectrometer (ESCALAB 250Xi). The vacuum degree of the analysis chamber was 8xl0-10Pa, the excitation source was Al ka ray (hv=1486.8eV), the working voltage was 12.5 kV, the filament current was 16 mA, and the signal accumulation was performed for 10 cycles. The passing energy was 30ev, the step size was 0.1eV, and the charge correction was performed using the binding energy of C1s=284.80eV as the energy standard.

**Gel Permeation Chromatography (GPC) Measurements**

The fibers were fully dissolved in a potassium hydroxide/DMSO solution (0.05 M KOH in anhydrous DMSO) and immediately analyzed by ambient-temperature gel-permeation chromatography (GPC) on an Agilent PL-GPC50 system equipped with two PLgel Mixed-B columns (300 mm × 7.5 mm, 10 µm particles) in series. The measurements were conducted at 50 °C with the eluent flowing at 1.0 mL min⁻¹.

**Small--angle X-ray scattering and wide-angle X-ray scattering (SAXS/WAXS) measurements**

SAXS/WAXS experiments were conducted on a laboratory-based Xeuss 2.0 instrument (Xenocs, France) equipped with a micro-focus Cu Kα source (30 W, λ = 1.54189 Å). A Pilatus 3R 300K detector (Dectris, Switzerland) with a pixel size of 172 × 172 μm and an active area of 83.8 × 33.5 mm was employed for 2D scattering pattern acquisition. The sample-to-detector distance (SDD) was calibrated at 1188 mm for SAXS and 86.5 mm for WAXS. The resulting analysis of the SAXS patterns and the WAXS patterns were performed by FIT two-dimensional (2D) software.

The arc deviation between the microfibril axis and the macroscopic fibre direction, herein termed the misorientation arc Bφ, was extracted from the SAXS equatorial streak via the Ruland streak analysis according to $s=\frac{2sin\theta}{\lambda}$ and $B_{obs}=\frac{1}{sl_{f}}+B_{\phi}$, where θ is the half value of the scattering angle, λ is the wavelength dimension of the X-ray, s is the scattering vector, Bobs is the full width at the half-maximum of the azimuthal profile, and l_f_ is the fibril length.

**Mechanical property tests of monofilament**

Quasi-static tensile measurements were performed with YG021G Pneumatic Electronic Fiber Strength Tester (Changzhou Shuanggu Dunda Electromechanical Technology Co., Ltd., China) at a speed of 1 mm min^-1^. A 200 mm single fiber was secured with pneumatic grips and vertically aligned between them. Fiber diameters were determined prior to testing by optical microscopy. Stress–strain curves were plotted in Origin 2024 from the recorded force–displacement data, and toughness was determined as the integrated area under each curve. Reported tensile strength, modulus, elongation at break, and toughness represent the mean ± standard deviation of ten valid specimens.

Stress-relaxation experiments on fibers were conducted using the DMA Q800 (TA, America). The test specimen was mounted on the sample stage with a gauge length of 10 mm. The initial strain of 0.75 % was applied and held constant for 5000 s to allow relaxation. Finally, a normalized stress–relaxation time curve was derived from the experimental data.

**Preparation and characterization of MXene-NH_2_**

As illustrated in Figure S1a, the as-prepared MXene nanosheets contain abundant hydroxyl (OH^-^) groups with negatively charged natures. When MXene nanosheets react with ammonia water under high temperature, the positively charged NH^4+^ groups from ammonia water will attack negatively charged OH^-^ groups on MXene surface, due to the low binding energy of OH^-^ groups on MXene surface.^[1,2]^ Accordingly, the OH^-^ groups are partially replaced by -NH_2_ groups to obtain MXene-NH_2_ nanosheets, which contain multitudinous active sites, such as oxygen-containing, fluorine-containing groups and active amino groups. Based on amination reaction with ammonia water, various MXene-NH_2_ nanosheets can be synthesized, such as such as Ti_3_C_2_T_x_-NH_2_, V_2_CT_x_-NH_2_, Nb_2_CT_x_-NH_2_ and Mo_2_TiC_2_T_x_-NH_2_ nanosheets. As a typical representative, the Ti_3_C_2_T_x_ MXene nanosheets are utilized as an example to conduct the structural and chemical characterization of MXene-NH_2_ nanosheets as well as subsequent PBIA/MXene-NH_2_ fiber. The lamellar structure of Ti_3_C_2_T_x_-NH_2_ nanosheets is first confirmed by scanning electron microscopy (SEM). Notably, the average lateral size of Ti_3_C_2_T_x_-NH_2_ nanosheets is about 0.75 µm with narrow size distribution at 0.6-1 µm (Figure S1b). The transmission electron microscope (TEM) image further reveals the typical 2D layered structure with single-layer morphology (Figure S1c). Moreover, the thickness of Ti_3_C_2_T_x_-NH_2_ nanosheets is characterized by atomic force microscope (AFM). As shown in Figure S1d, the Ti_3_C_2_T_x_-NH_2_ nanosheets possess a single-layer thickness of 1.6 nm and a lateral size of 0.8 µm, which is highly consistent with SEM and TEM results.

**
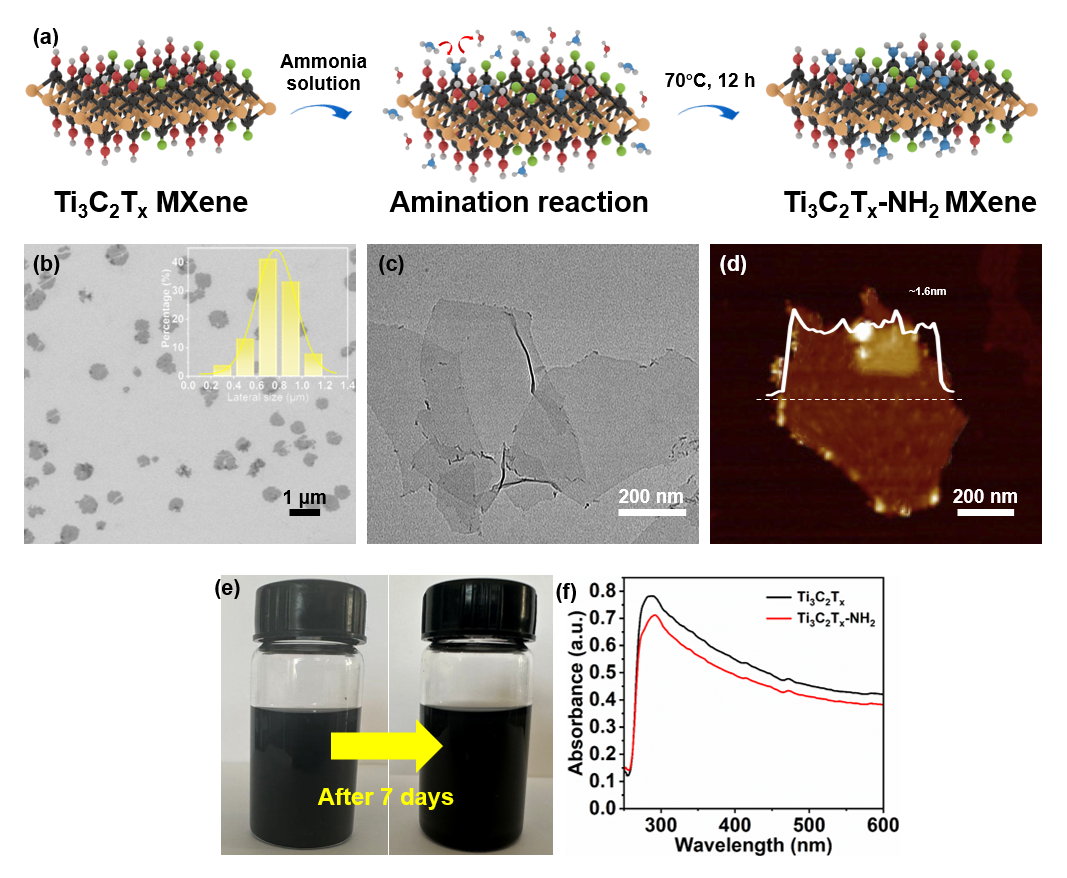
**

**Figure S1.** (a) Schematic synthesis of MXene-NH_2_ nanosheets. (b) The SEM image of Ti_3_C_2_T_x_-NH_2_ nanosheets. (c) The TEM image of Ti_3_C_2_T_x_-NH_2_ nanosheets. (d) The AFM image of Ti_3_C_2_T_x_-NH_2_ nanosheets. (e) The digital images of Ti_3_C_2_T_x_ dispersed in DMAc on the first and seventh days. f) The UV-vis absorption curves of Ti_3_C_2_T_x_/DMAc and Ti_3_C_2_T_x_ -NH_2_/DMAc solution.

The chemical environments of Ti_3_C_2_T_x_ and Ti_3_C_2_T_x_-NH_2_ nanosheets are further investigated to confirm the successful grafting of -NH_2_ groups on Ti_3_C_2_T_x_-NH_2_ nanosheets. Figure S1a depicts the Fourier-transform infrared (FTIR) spectroscopy of Ti_3_C_2_T_x_ and Ti_3_C_2_T_x_-NH_2_ nanosheets. Compared to Ti_3_C_2_T_x_ nanosheets, the Ti_3_C_2_T_x_-NH_2_ nanosheets emerge new characteristic peaks of -NH_2_ groups at 670 cm^-1^, 1256 cm^-1^ and 3436 cm^-1^, corresponding to Ti-N, C-N and N-H chemical bonds, respectively.^[2]^ ^[3]^ Moreover, the Raman spectra of Ti_3_C_2_T_x_-NH_2_ nanosheets also present unique characteristic peaks of C-N chemical bonds at 1271 cm^-1^ and lower I_D_/I_G_ ratio than that of Ti_3_C_2_T_x_ nanosheets, indicating the successful grafting of -NH_2_ groups on Ti_3_C_2_T_x_-NH_2_ nanosheets (Figure S2b). The X-ray diffraction (XRD) analysis is illustrated in Figure S2c. Obviously, the (002) characteristic peak of Ti_3_C_2_T_x_-NH_2_ nanosheets decrease from 6.67º to 6.22º when compared with Ti_3_C_2_T_x_ nanosheets, reflecting that the -NH_2_ groups are grafted into Ti_3_C_2_T_x_-NH_2_ nanosheets interlayer and therefore increase the interlayer spacin.^[4]^ The specific chemical groups and bonds are validated by X-ray photoelectron spectroscopy (XPS) analysis. Different from Ti_3_C_2_T_x_ nanosheets that only contain C, Ti, O and F elements, the XPS survey spectra of Ti_3_C_2_T_x_-NH_2_ nanosheets contains C, N, Ti, O and F elements, implying the existence of -NH_2_ groups (Figure S2d). As illustrated in Figure S2e, the high-resolution N 1s spectrum has five main peaks at 396.4, 399.2, 400.4, 401.8 and 402.8 eV, corresponding to N-Ti, -NH_2_, -NH_4_^+^, N-C and -NH_3_^+^, which demonstrates the successful synthesis of Ti_3_C_2_T_x_-NH_2_ nanosheets via amination reaction.^[2]^

**
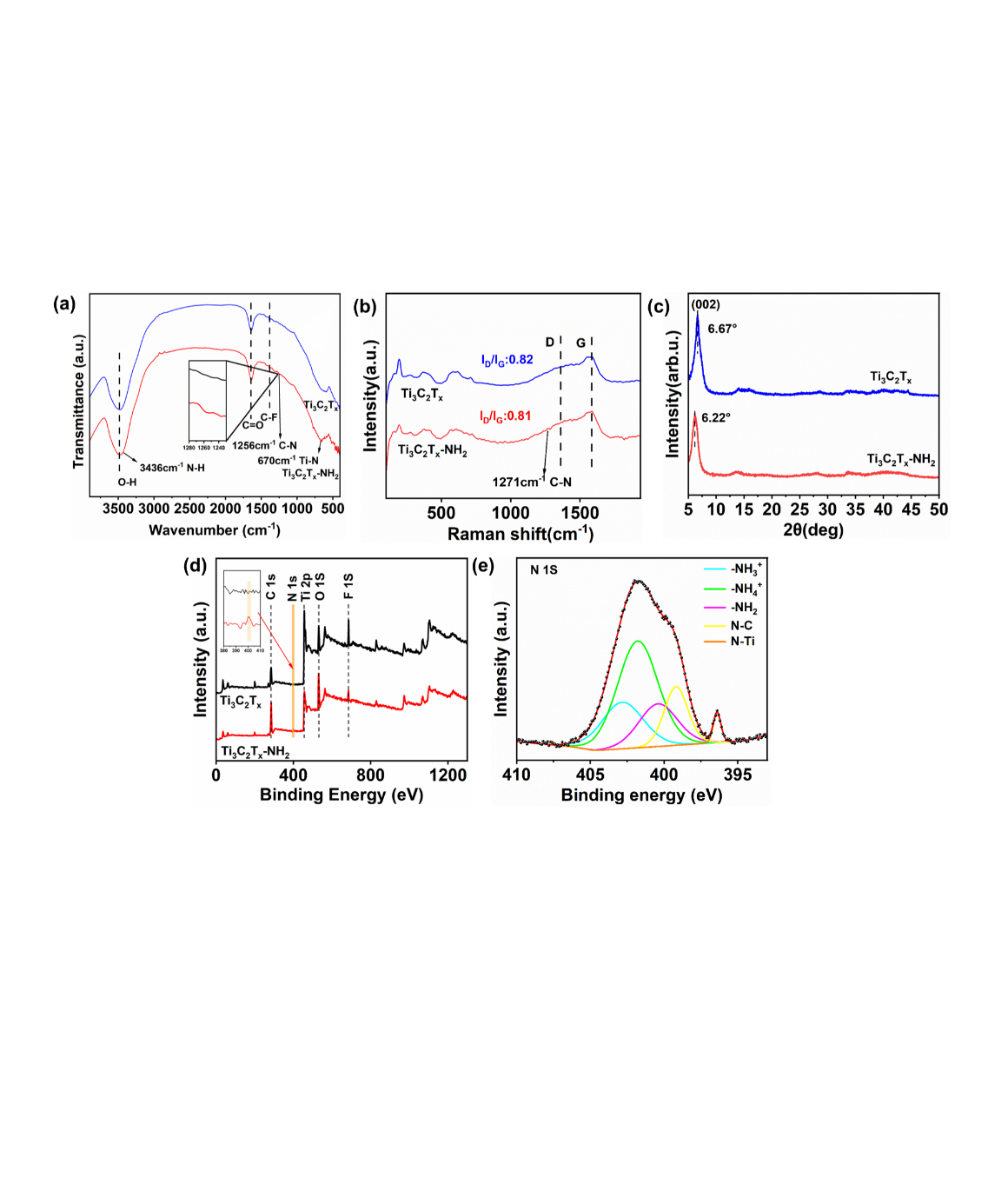
**

**Figure S2.** (a) The FT-IR spectra of Ti_3_C_2_T_x_ and Ti_3_C_2_T_x_-NH_2_ nanosheets. (b) The Raman spectra of of Ti_3_C_2_T_x_ and Ti_3_C_2_T_x_-NH_2_ nanosheets. (c) The XRD spectra of Ti_3_C_2_T_x_ and Ti_3_C_2_T_x_-NH_2_ nanosheets. (d) The XPS survey spectra of Ti_3_C_2_T_x_ and Ti_3_C_2_T_x_-NH_2_ nanosheets. (e) The high-resolution XPS N 1s spectrum of Ti_3_C_2_T_x_-NH_2_ nanosheets.

**Supplementary Figures**

**
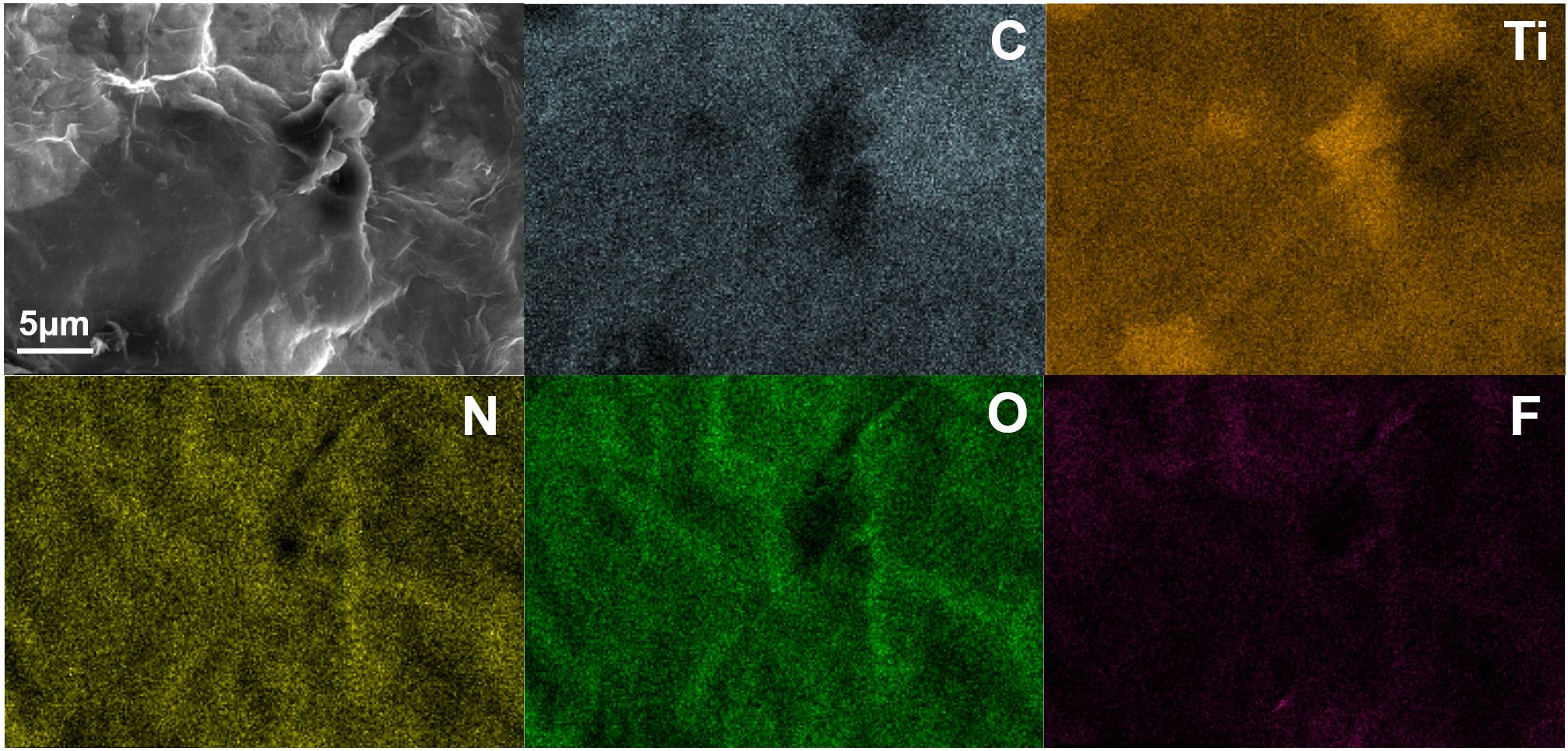
**

**Figure S3.** SEM-EDS elemental mapping images of Ti_3_C_2_T_x_-NH_2_ nanosheets

**
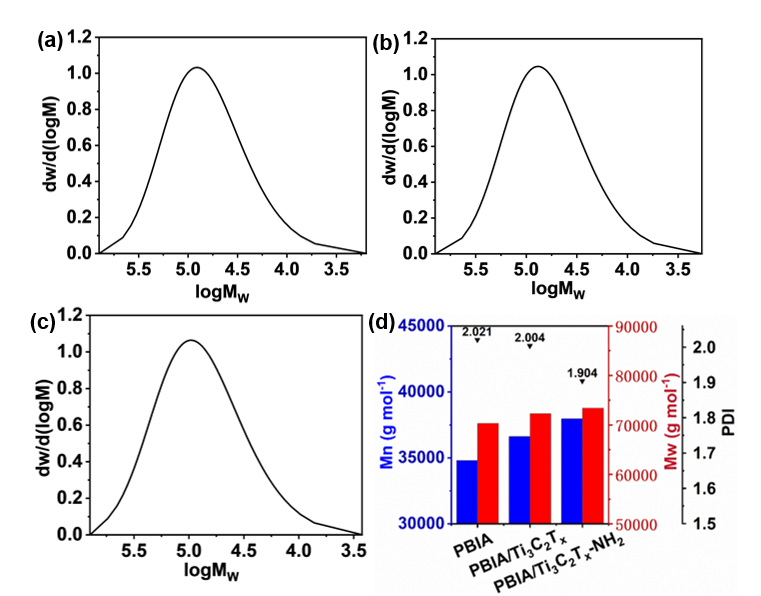
**

**Figure S4.** (a) The chromatogram of polymer solutions of PBIA. (b) The chromatogram of polymer solutions of PBIA/Ti_3_C_2_T_x_. (c) The chromatogram of polymer solutions of PBIA/Ti_3_C_2_T_x_-NH_2_ (d) The GPC measurements of PBIA spinning dope, PBIA/Ti_3_C_2_T_x_ spinning dope and PBIA/Ti_3_C_2_T_x_-NH_2_ spinning dope.

**
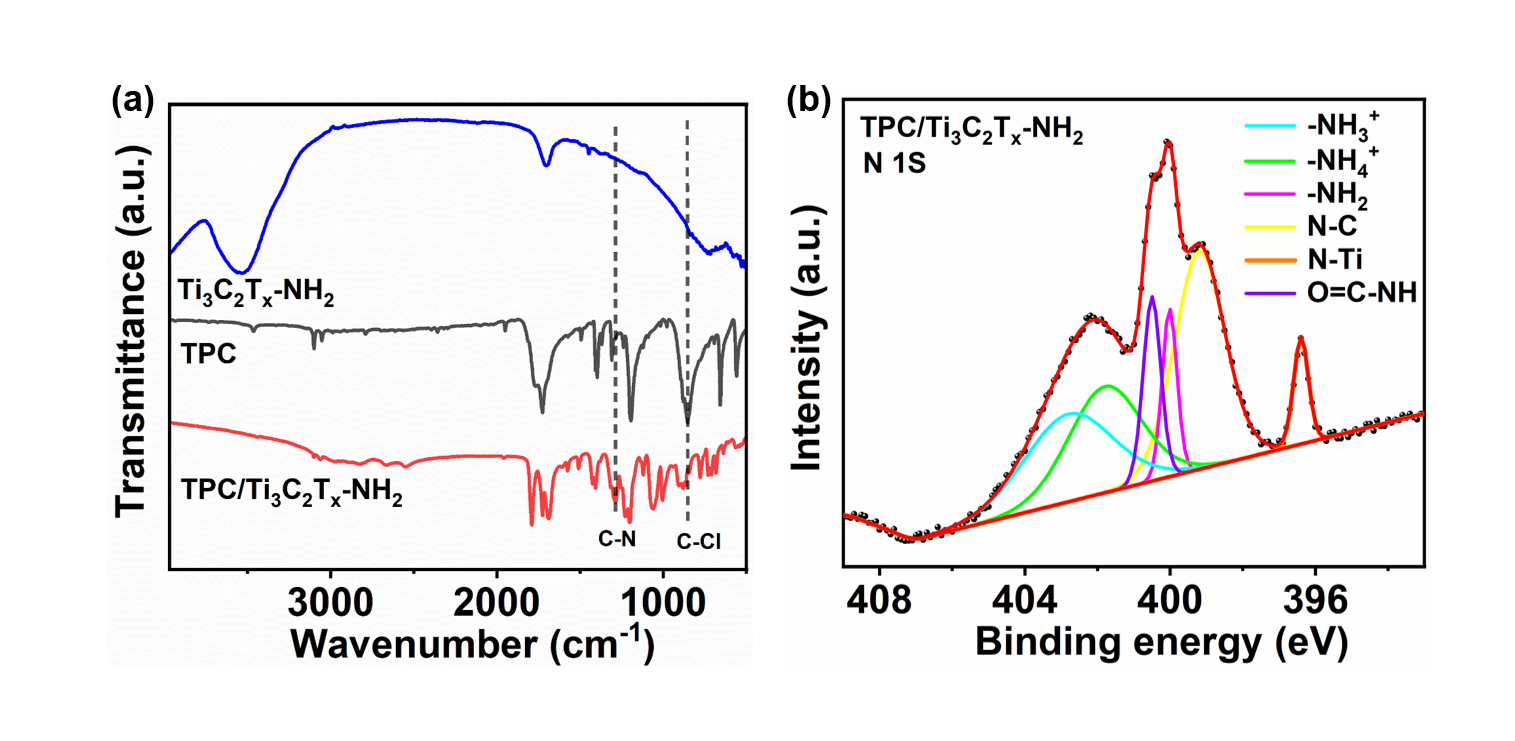
**

**Figure S5.** (a) The FTIR spectra of Ti_3_C_2_T_x_-NH_2_, TPC and TPC/Ti_3_C_2_T_x_-NH_2_. (b) The high-resolution XPS N 1s spectrum of the TPC/Ti_3_C_2_T_x_-NH_2_ nanosheets

**

**

**Figure S6.** Comparison of key parameters between PBIA fibers and PBIA/Ti₃C₂Tₓ-NH₂ fibers (0.025 wt%)

**

**

**Figure S7.** The FTIR spectra of PBIA fibers, PBIA/Ti_3_C_2_T_x_ fibers and PBIA/Ti_3_C_2_T_x_-NH_2_ fibers.

**
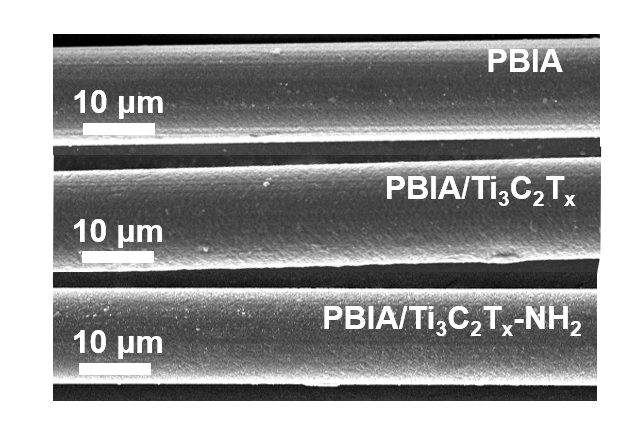
**

**Figure S8.** The SEM images of PBIA fibers, PBIA/Ti_3_C_2_T_x_ fibers and PBIA/Ti_3_C_2_T_x_-NH_2_ fibers.

**
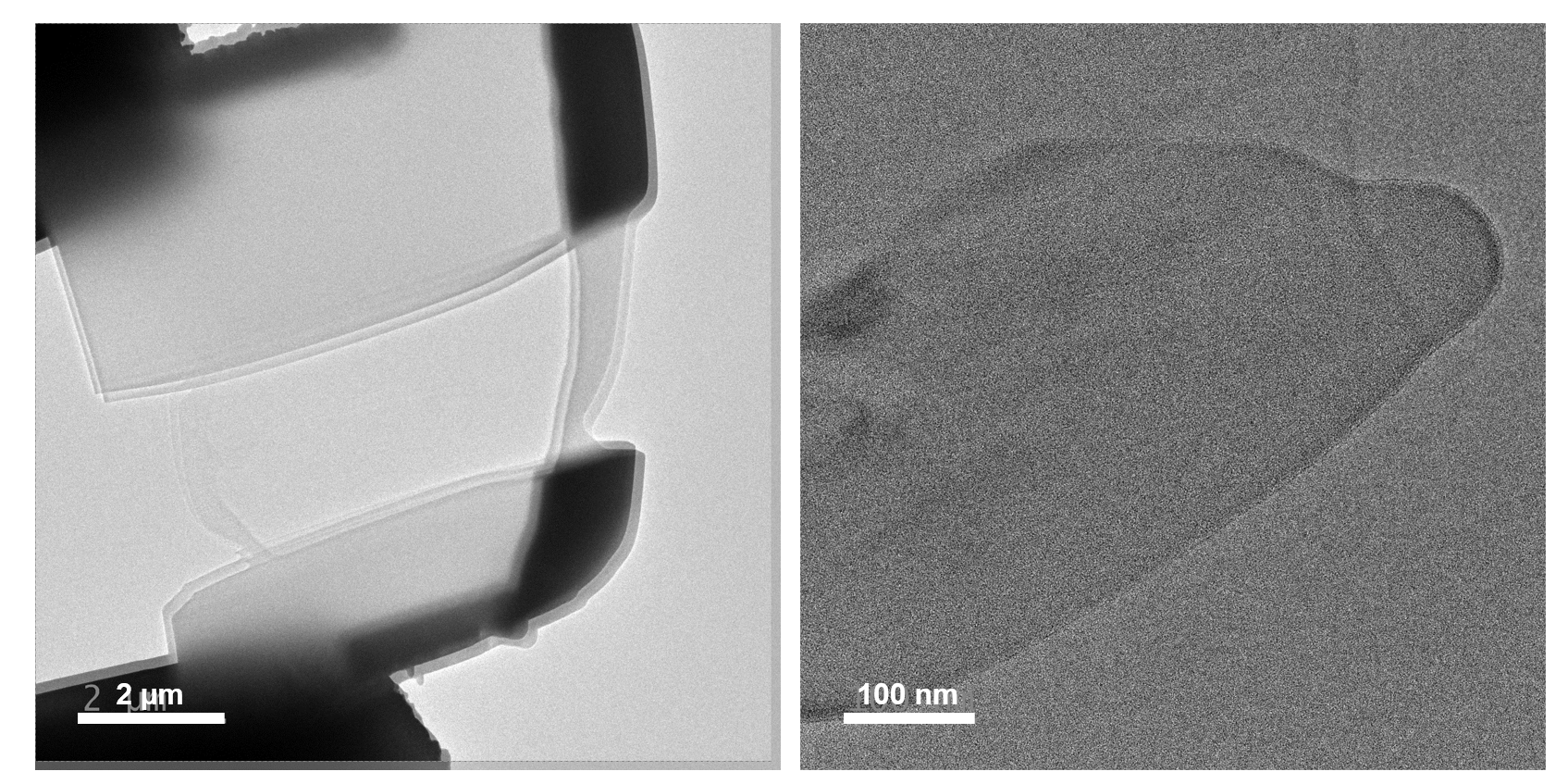
**

**Figure S9.** The TEM images of PBIA/Ti_3_C_2_T_x_-NH_2_ fibers prepared by FIB.


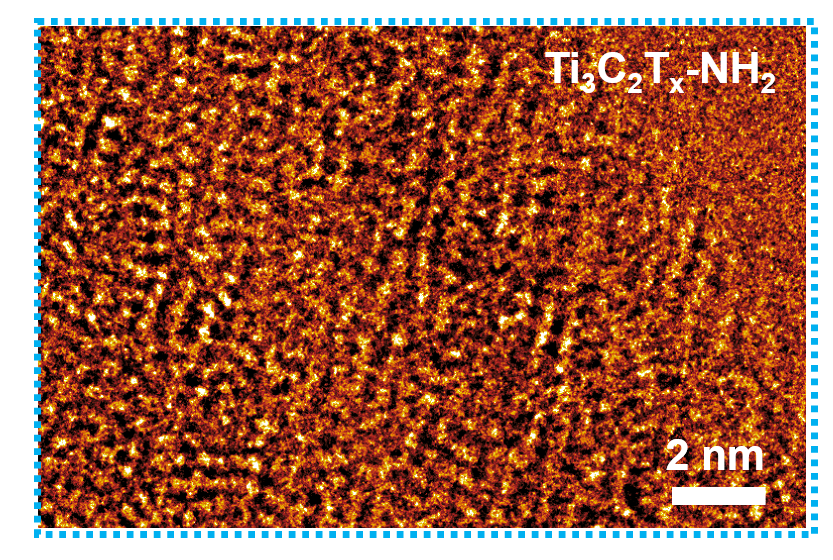


**Figure S10.** The AC-TEM images of PBIA/Ti_3_C_2_T_x_-NH_2_ fiber.

**

**

**Figure S11.** The 1D-WAXS curve of PBIA fibers, PBIA/Ti_3_C_2_T_x_ fibers and PBIA/Ti_3_C_2_T_x_-NH_2_ fibers.

**

**

**Figure S12.** The 1D-WAXS curves and peak fitting curves for PBIA fibers.

**
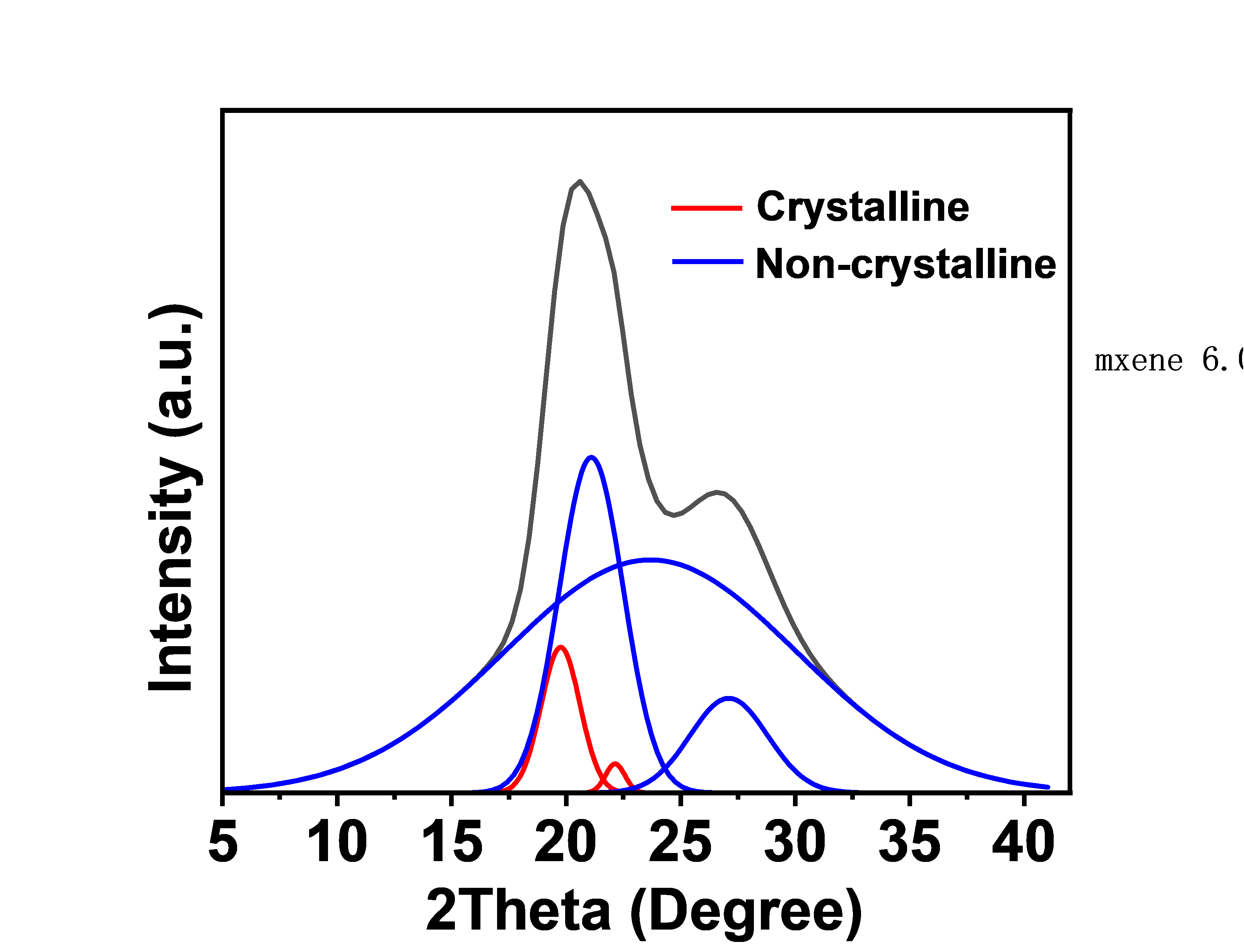
**

**Figure S13.** The 1D-WAXS curves and peak fitting curves for PBIA/Ti_3_C_2_T_x_ fibers.

**
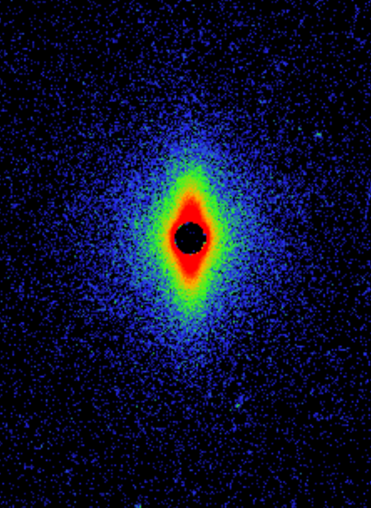
**

**Figure S14.** The 2D-SAXS spectrum of PBIA fibers.

**
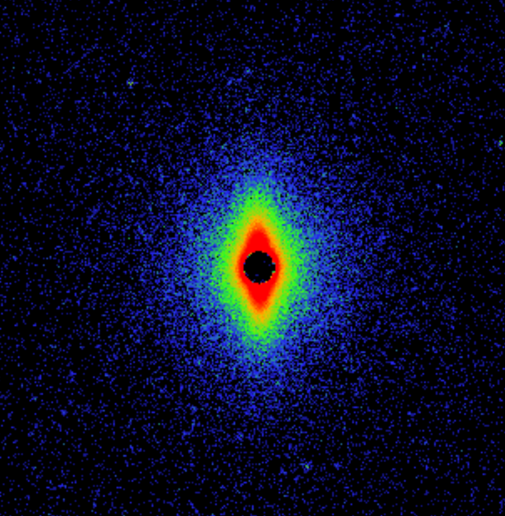
**

**Figure S15.** The 2D-SAXS spectrum of 0.025wt% added PBIA/Ti_3_C_2_T_x_ fibers.

**
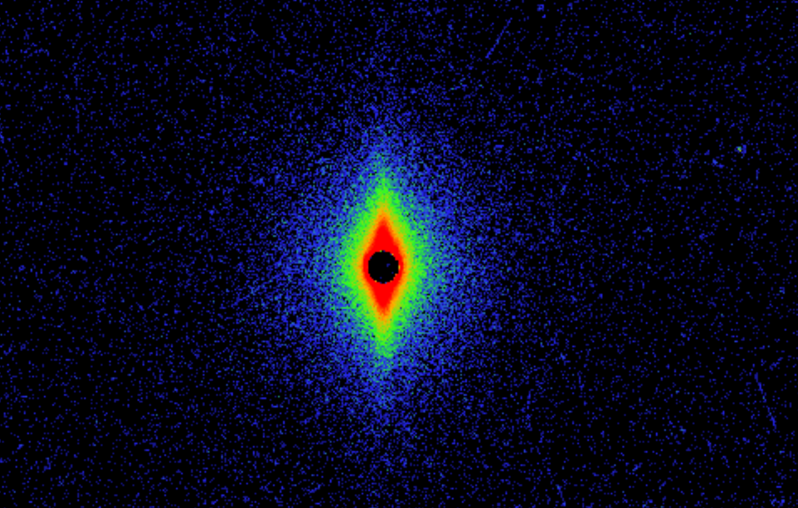
**

**Figure S16.** The 2D-SAXS spectrum of 0.025wt% added PBIA/Ti_3_C_2_T_x_-NH_2_ fibers.





**Figure S17.** The corresponding Ruland plot for PBIA (Bobs vs. q^-1^, where Bobs is the full width at half maximum of the azimuthal scan).





**Figure S18.** The corresponding Ruland plot for PBIA/Ti_3_C_2_T_x_ (Bobs vs. q^-1^, where Bobs is the full width at half maximum of the azimuthal scan).





**Figure S19.** The corresponding Ruland plot for PBIA/Ti_3_C_2_T_x_-NH_2_ (Bobs vs. q^-1^, where Bobs is the full width at half maximum of the azimuthal scan).

**

**

**Figure S20.** The misorientation degree of PBIA, PBIA/Ti_3_C_2_T_x_ and PBIA/Ti_3_C_2_T_x_-NH_2_ fibers.

**
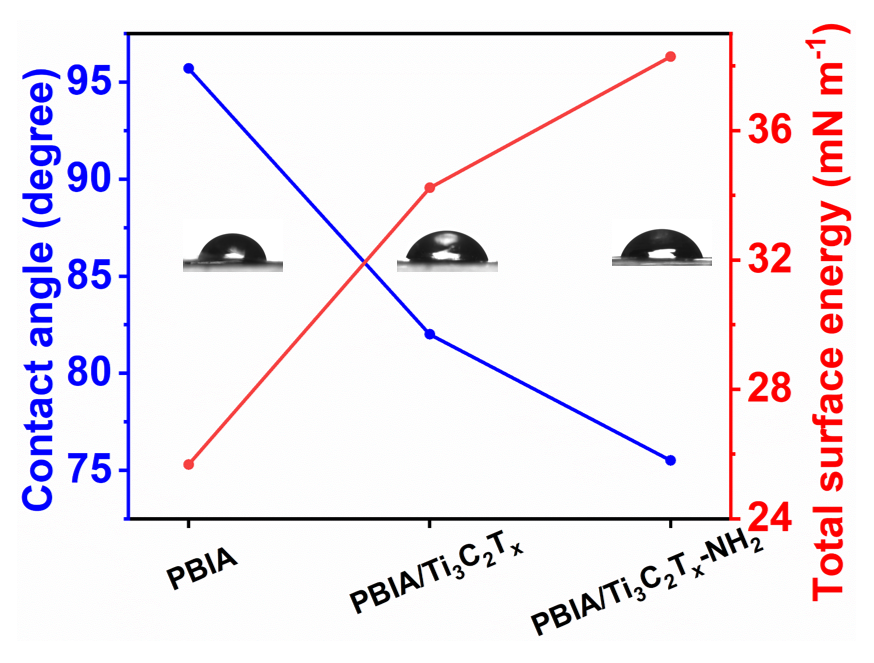
**

**Figure S21.** Comparison of the contact angle and total surface energy of different fibers

**
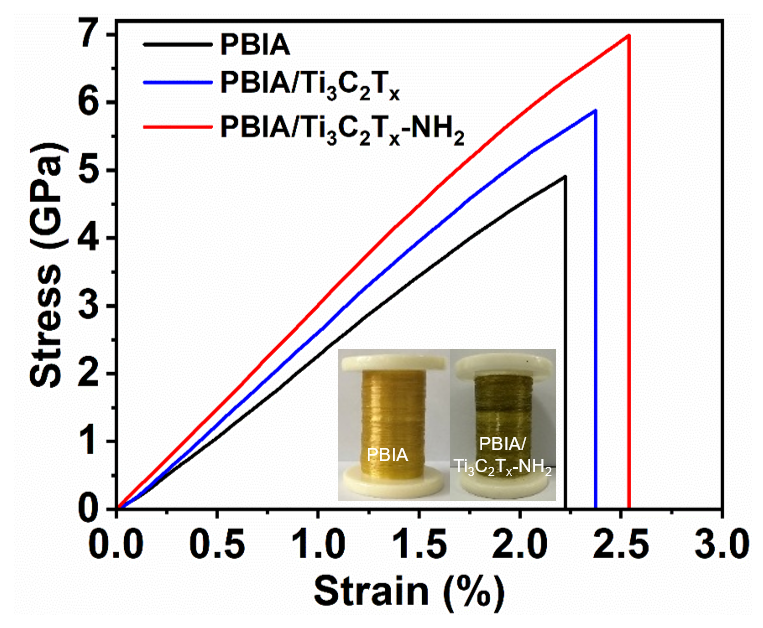
**

**Figure S22.** The stress-strain curves of PBIA, PBIA/Ti_3_C_2_T_x_ and PBIA/Ti_3_C_2_T_x_-NH_2_ fibers, the inset: digital photographs of PBIA and PBIA/Ti_3_C_2_T_x_-NH_2_ fibers.

**

**

**Figure S23.** The tensile strength and Young’s modulus of PBIA and PBIA/Ti_3_C_2_T_x_-NH_2_ fibers before and after bending fatigue tests.

**

**

**Figure S24.** The tensile strength and Young’s modulus of PBIA and PBIA/Ti_3_C_2_T_x_-NH_2_ fibers before and after knot strength tests.

**

**

**Figure S25.** The tensile strength and Young’s modulus of PBIA and PBIA/Ti_3_C_2_T_x_-NH_2_ fibers before and after hygrothermal aging tests.

**
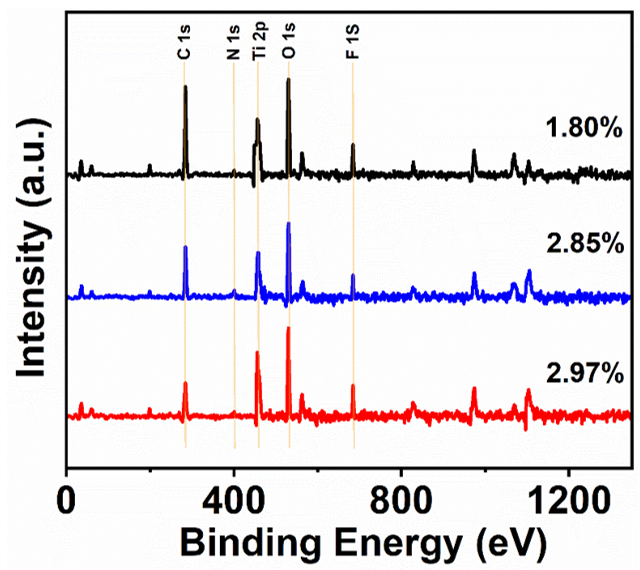
**

**Figure S26.** The -NH_2_ contents of various Ti_3_C_2_T_x_-NH_2_ nanosheets under various ammonia water concentration.

**
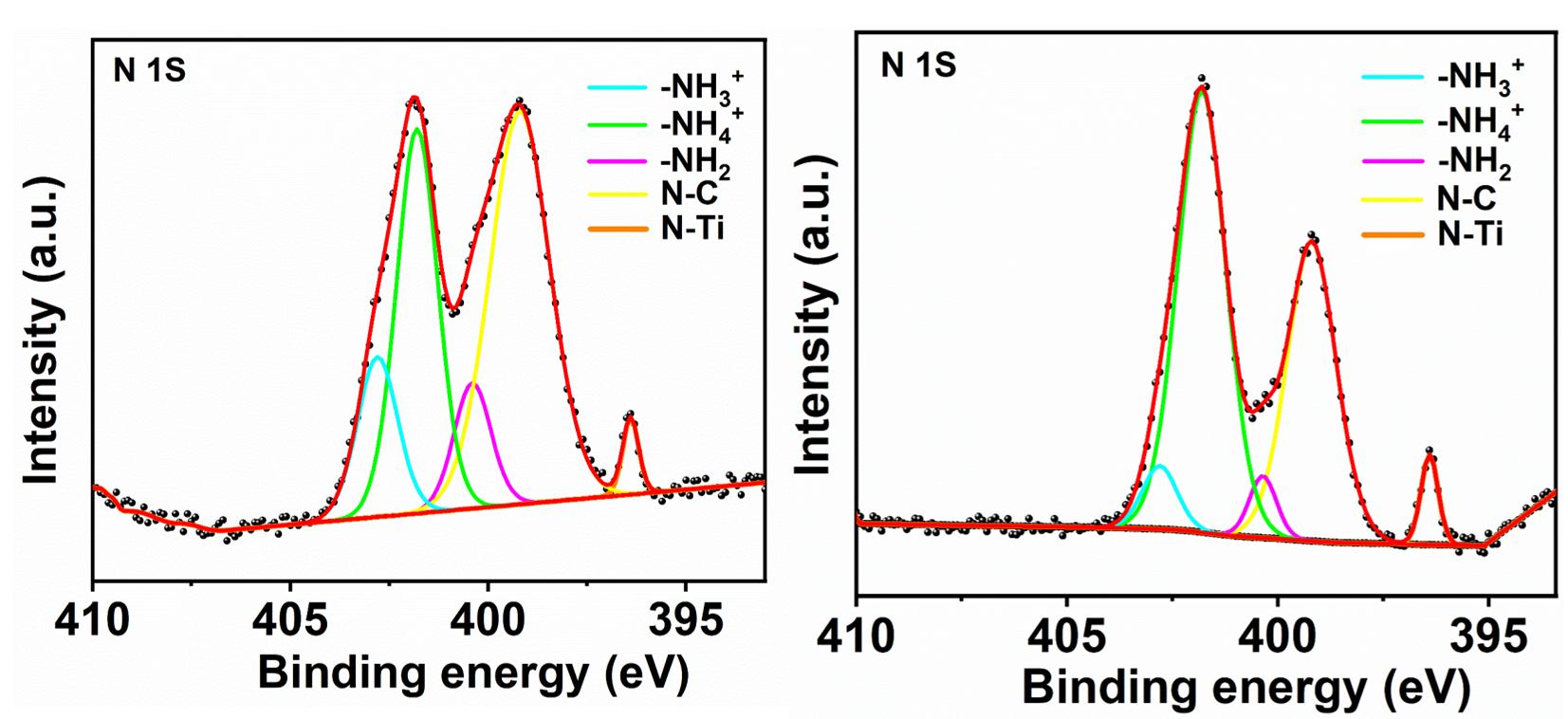
**

**Figure S27.** The high-resolution N 1s spectrum of Ti_3_C_2_T_x_-NH_2_-1.80% and Ti_3_C_2_T_x_-NH_2_-2.97%.

**
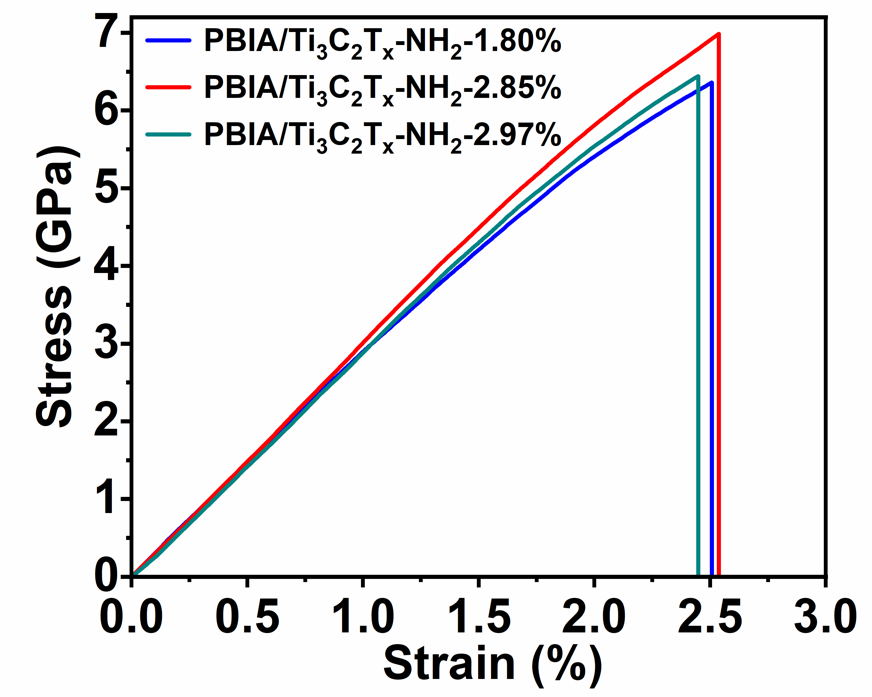
**

**Figure S28.** The stress-strain curves of PBIA/Ti_3_C_2_T_x_-NH_2_ fibers under various Ti_3_C_2_T_x_-NH_2_ nanosheets with various -NH_2_ contents.

**
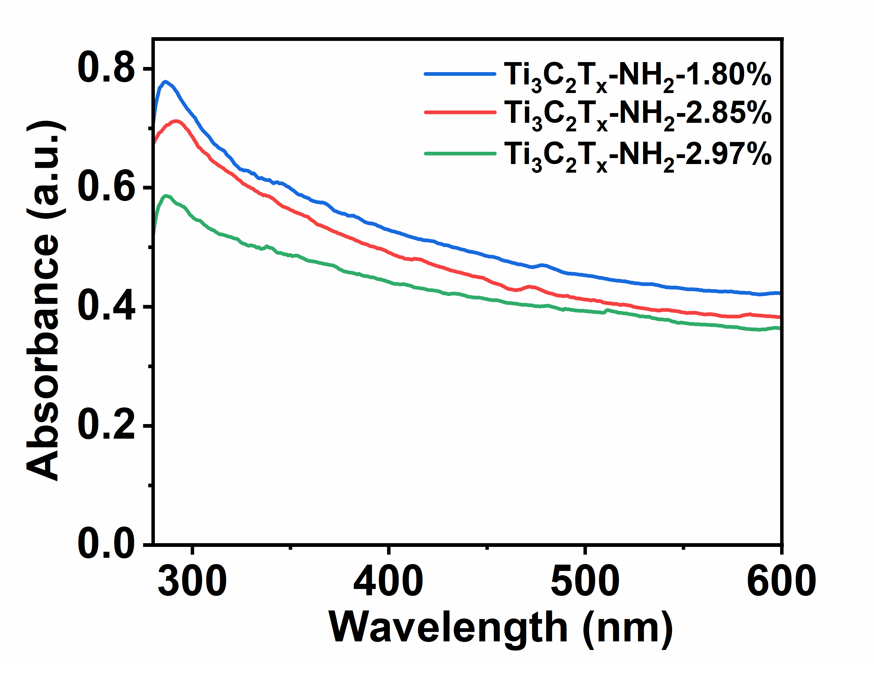
**

**Figure S29.** The UV–vis absorption curves of Ti_3_C_2_T_x_ -NH_2_-1.80%/DMAc, Ti_3_C_2_T_x_ -NH_2_-2.85%/DMAc and Ti_3_C_2_T_x_ -NH_2_-2.97%/DMAc solution.


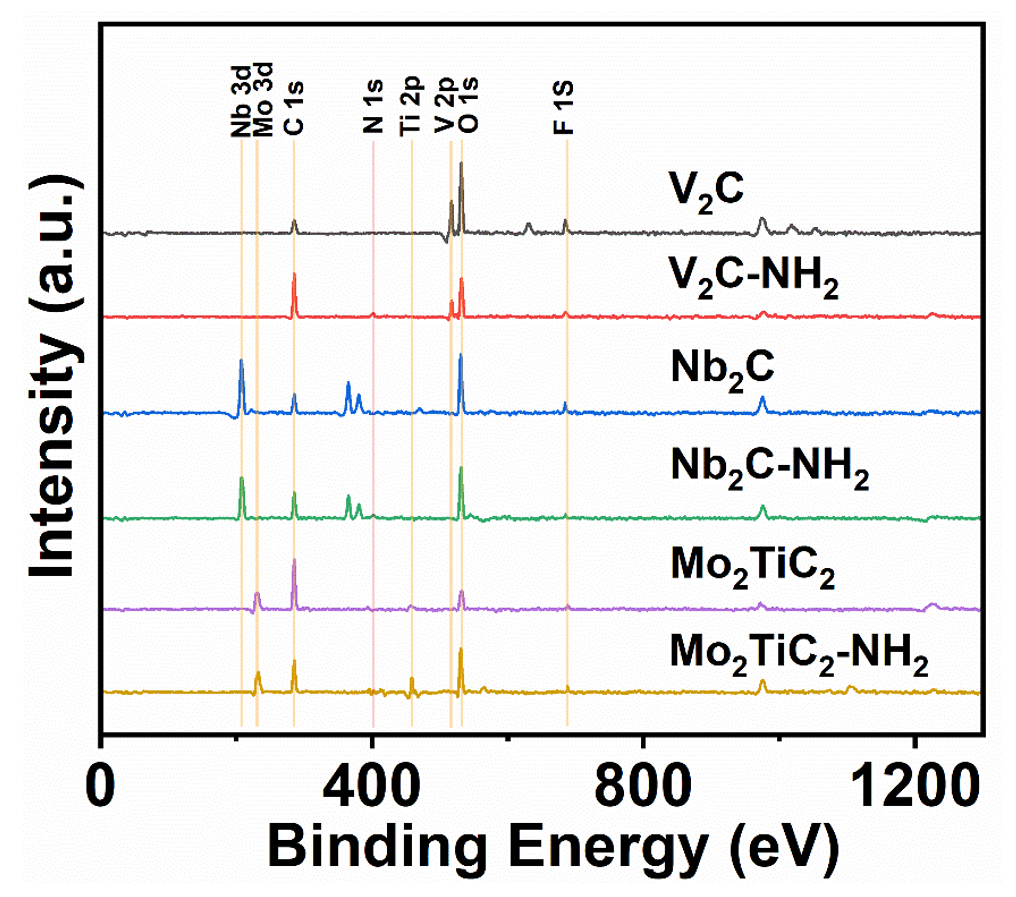


**Figure S30.** The XPS survey spectra of V_2_CT_x_, Nb_2_CT_x_, Mo_2_TiC_2_T_x_, V_2_CT_x_-NH_2_, Nb_2_CT_x_-NH_2_ and Mo_2_TiC_2_T_x_-NH_2_ nanosheets.


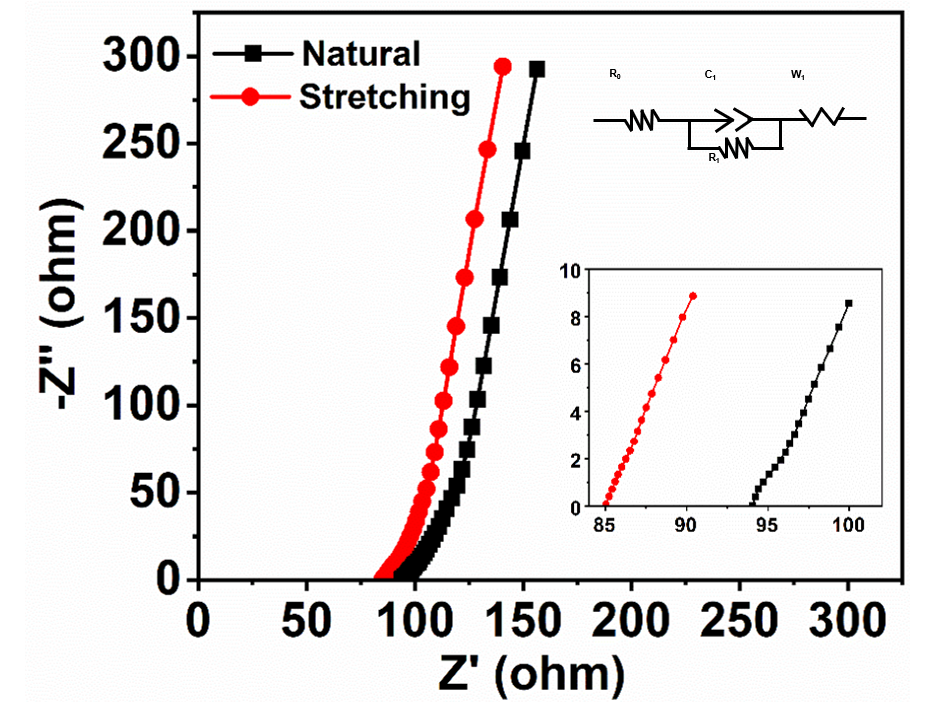


**Figure S31.** The Nyquist plots of CNTs@PBIA/Ti_3_C_2_T_x_-NH_2_ FSC under natural and stretching states, the inset: equivalent circuit model.


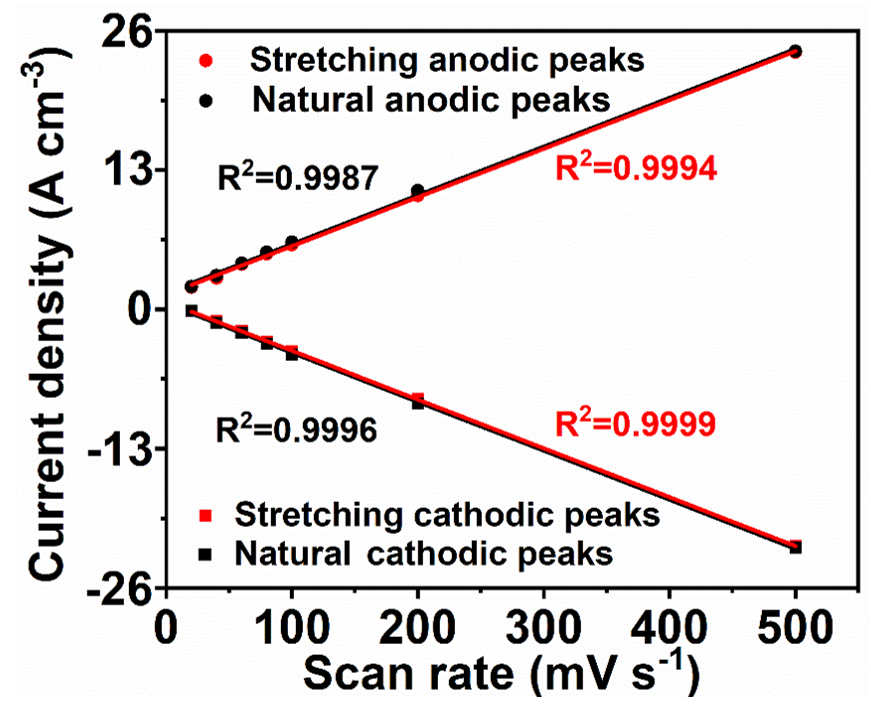


**Figure S32.** The current densities versus scan rates of CNTs@PBIA/Ti_3_C_2_T_x_-NH_2_ FSC under natural and stretching states.

**Table S1. Mechanical properties of PBIA fibers**

| Number | Tensile strength (GPa) | Modulus (GPa) |
| --- | --- | --- |
| 1 | 4.98 | 228.2 |
| 2 | 5.30 | 234.5 |
| 3 | 5.07 | 232.6 |
| 4 | 5.22 | 232.7 |
| 5 | 5.23 | 234.0 |
| Average | 5.16 | 232.4 |

**Table S2. Mechanical properties of PBIA/Ti_3_C_2_T_x_-NH_2_ fibers**

| Number | Tensile strength (GPa) | Modulus (GPa) |
| --- | --- | --- |
| 1 | 6.63 | 270.6 |
| 2 | 7.27 | 275.7 |
| 3 | 7.20 | 271.3 |
| 4 | 7.25 | 273.8 |
| 5 | 6.40 | 276.6 |
| Average | 6.95 | 273.6 |

**References**

(1)X. Sang, et al., “Unocic, Atomic Defects in Monolayer Titanium Carbide (Ti_3_C_2_T_x_) MXene,” *ACS Nano* 10, (2016**)**: 9193.

10.1021/acsnano.6b05240

(2)M. Peng, et al., “Amino Termination of Ti_3_C_2_ MXene Induces its Graphene Hybridized Film with Enhanced Ordered Nanostructure and Excellent Multiperformance,” *Advanced Materials Interfaces* 9. (2022**)**: 2102418.

<https://doi.org/10.1002/admi.202102418>

(3)M. Q. Snyder, et al., “An infrared study of the surface chemistry of titanium nitride atomic layer deposition on silica from TiCl_4_ and NH_3_,” *Thin Solid Films* 514, (2006**)**: 97.

<https://doi.org/10.1016/j.tsf.2006.03.013>

(4)J. Wan, R. et al., “Amino modification of Ti_3_C_2_ MXenes for high-performance supercapacitors,” *Applied Surface Science* 678, (2024): 161154.

<https://doi.org/10.1016/j.apsusc.2024.161154>
